# Synthesis, characterization, X-ray diffraction, *in silico* studies and *in vitro* biological evaluation of acylthiourea-derived thiazole-2-imines

**DOI:** 10.1039/d6ra07024h

**Published:** 2026-10-08

**Authors:** Edmore F. Kangara, Thishana Singh, Phindile Khoza, Darian Naidu, Nompumelelo P. Mkwananzi, Sibusiso Senzani, Oluwatoba E. Oyeneyin, Manuel A. Fernandes, Werner Van Zyl, Matshawandile Tukulula

**Affiliations:** a Molecular Sciences Institute, School of Chemistry, University of the Witwatersrand Private Bag X3 Johannesburg 2050 South Africa kangara.edd@gmail.com; b Discipline of Chemistry, School of Agriculture and Engineering, University of KwaZulu-Natal Private Bag X54001, Westville Durban 4000 South Africa TukululaM@ukzn.ac.za; c HIV Pathogenesis Programme, Doris Duke Medical Research Institute, School of Laboratory Medicine and Medical Sciences, College of Health Sciences, University of KwaZulu-Natal Durban 4001 South Africa; d School of Laboratory Medicine and Medical Science, College of Health Sciences, University of KwaZulu-Natal Medical School Campus Durban 4001 South Africa; e Department of Chemical and Physical Sciences, Walter Sisulu University (WSU) Private Bag X01 Mthatha Eastern Cape 4099 South Africa

## Abstract

A series of thiazole-2-imines (1a–1f) with unsubstituted aryl groups or aryl groups bearing methyl, methoxy, fluoro, or nitro substituents was designed using selected structural features of thiamine as inspiration. The compounds were synthesized from monosubstituted acylthioureas and *para*-substituted phenacyl bromides in 82–95% yields. Various spectroscopic techniques and high-resolution mass spectrometric analysis confirmed product formation. Single-crystal X-ray diffraction of the methyl/methyl, fluoro/fluoro, and nitro/fluoro-substituted derivatives established the *Z*-configured scaffold. Density functional theory (DFT) calculations supported the observed experimental geometries. Flexophore analysis indicated low-to-moderate similarity to thiamine, with the nitro/fluoro-substituted derivative yielding the highest score (41%). Docking of hypothetical pyrophosphorylated derivatives and the parent thiazole-2-imines suggested that these compounds could be accommodated within selected binding pockets of thiamine-dependent enzymes and TB- or HIV-related proteins. However, these predictions do not establish antimetabolite activity or specific molecular targets. *In silico* absorption, distribution, metabolism, excretion, and toxicity profiling predicted no Lipinski violations, low metabolic liability, and at least 74% oral absorption. *In vitro* evaluation against *Mycobacterium tuberculosis* H37Rv showed modest antimycobacterial activity, with MIC_90_ values ranging from 125 to 250 µM. The nitro/fluoro substituted derivative (1f) showed the strongest anti-HIV activity (IC_50_ = 75.86 µM) and the most favourable HIV-specific selectivity index, followed by the methyl/methyl-substituted derivative (1b).

## Abstract 2

A series of thiazole-2-imines (1a–1f) with unsubstituted aryl groups or aryl groups bearing methyl, methoxy, fluoro, or nitro substituents was designed using selected structural features of thiamine as inspiration.

## Graphical abstract

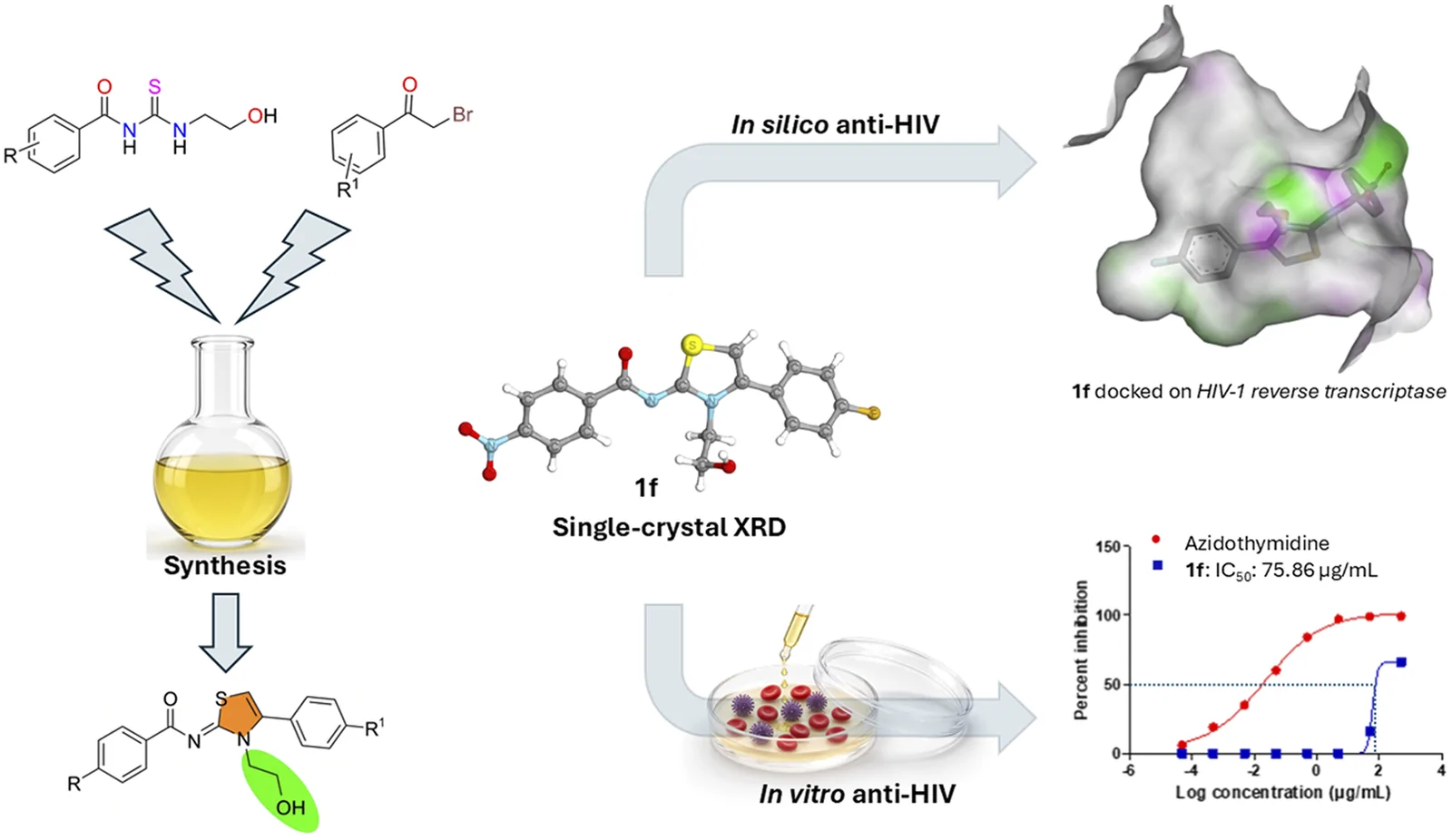

## Introduction

Thiazole-based compounds have long intrigued chemists and biologists alike, owing to their pivotal roles in biological processes and therapeutic applications. Among naturally occurring thiazole-containing molecules, thiamine (vitamin B1) is particularly important, functioning as the precursor of thiamine pyrophosphate, an essential cofactor in central metabolic pathways.^1–3^ The thiazole motif is also present in several approved drugs, highlighting its value as a privileged scaffold in medicinal chemistry (Fig. 1).^4–6^ Thiazole-2-imines derived from acylthioureas are of interest because they combine a thiazole-based scaffold with tunable substituents and are associated with antimicrobial, anti-human immunodeficiency virus (HIV), anticancer, and enzyme-inhibitory activities.^7–10^

**Fig. 1:**
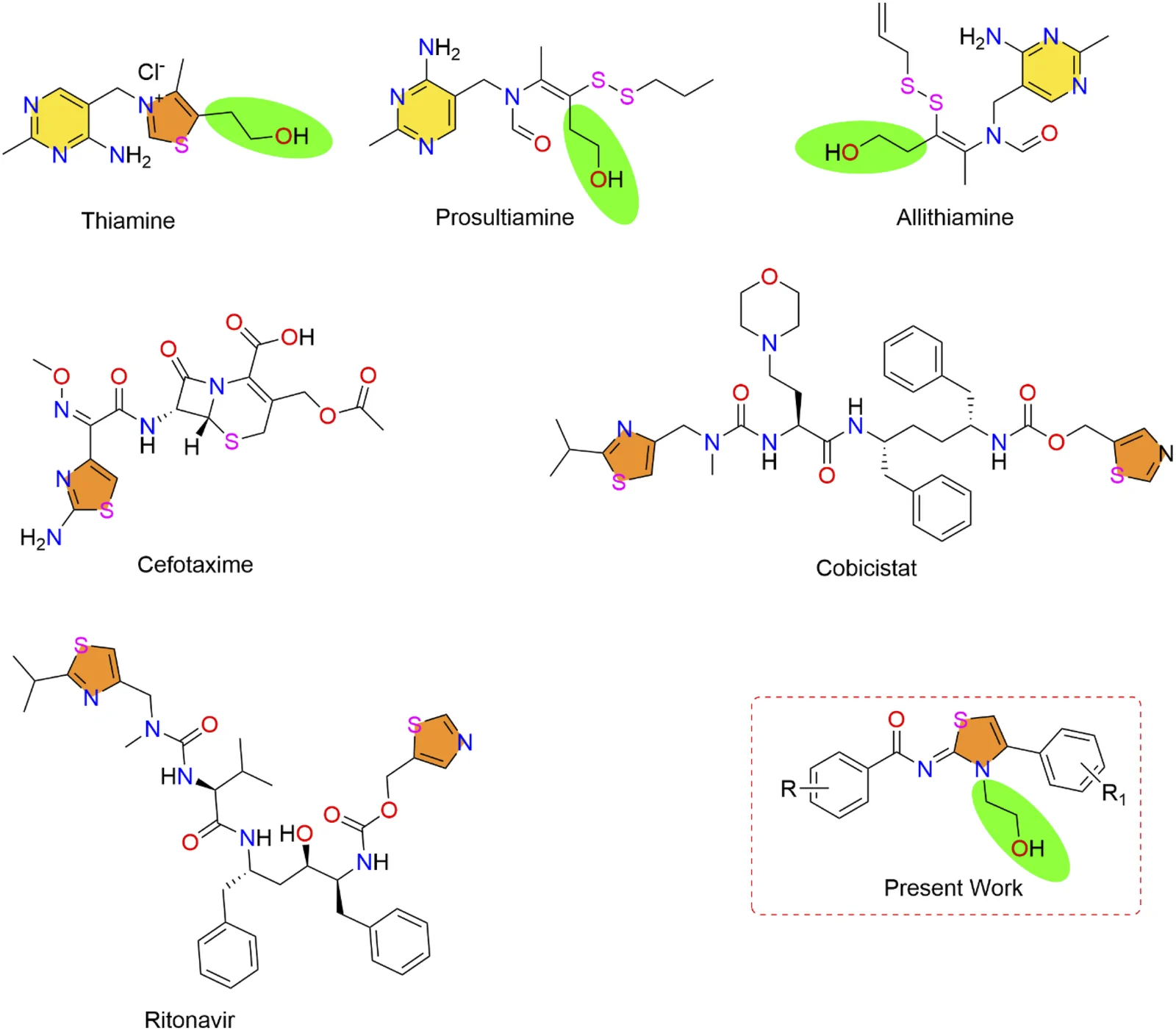
Structures of thiamine, thiamine analogues, and some antibacterial and antiviral drugs highlighting the thiazole ring and the pendant hydroxyethyl arm as structural features that inspired this work.

The global burden of tuberculosis (TB) and HIV, together with the emergence of multidrug-resistant strains, highlights the need for new chemical entities with alternative mechanisms of action.^11^ This is especially crucial in TB/HIV co-infection, where overlapping disease biology and treatment challenges complicate therapeutic interventions.^12^ One strategy for identifying new anti-infective candidates involves designing small molecules inspired by essential metabolites or cofactors, to disrupt pathogen metabolism through competitive- or antimetabolite mechanisms.^13^

In this study, acylthiourea-derived thiazole-2-imines were synthesized as putative anti-TB and anti-HIV agents. These compounds were designed to exploit some structural features of the thiazole-containing vitamin B_1_ framework, while enabling electronic modulation *via para*-substituted aryl groups. In pathogens, thiamine-dependent enzymes and associated metabolic pathways are essential for energy production, biosynthetic processes, survival, and virulence.^14–16^ Therefore, molecules containing key thiamine recognition elements may disrupt normal cofactor function, interfering with pathogen metabolism. This approach may provide a useful platform for exploring new anti-infective leads.

Herein, we report the synthesis and structural characterization of a series of thiazole-2-imine derivatives, supported by spectroscopic analysis, single-crystal X-ray diffraction, and DFT calculations. Their structural similarity to thiamine was assessed using flexophore descriptor analysis. Molecular docking was used to explore potential interactions with thiamine-dependent enzymes and selected TB- and HIV-related targets. Additionally, *in silico* ADME/T profiling and *in vitro* antitubercular, anti-HIV, and TZM-bl cell-based cytotoxicity studies were performed to evaluate their preliminary drug-like and biological activities.

## Results and discussion

### Synthesis

Thiazole-2-imine derivatives (1a–1f) were obtained *via* the cyclization of monosubstituted acylthioureas using *para*-substituted phenacyl bromides (Scheme 1). Monosubstituted acylthioureas were synthesized as reported in the literature.^17,18^ The thiazole-2-imine derivatives were then synthesized in excellent yields, *via* the classical *in situ* Hantzsch thiazole synthesis,^19^ by reacting a monosubstituted acylthiourea with the corresponding *para*-substituted phenacyl bromide in acetone at ambient temperature for 12 h. The R and R^1^ substituents were two electron-withdrawing groups (–NO_2_ and –F), and three electron-donating groups (–H, –Me, and –MeO), respectively.

**Scheme 1:**
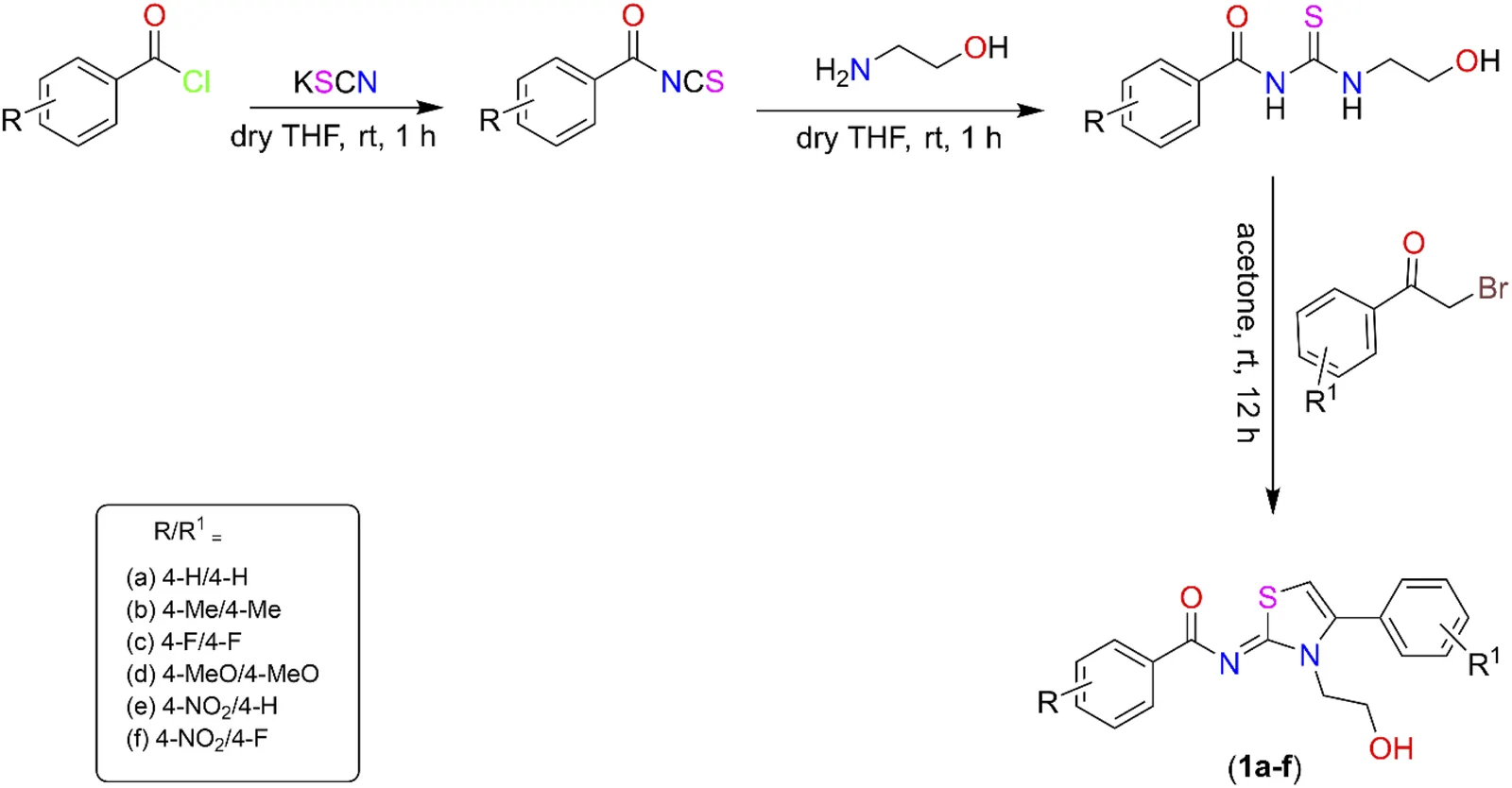
Synthesis of compounds 1a–1f.

### Spectroscopic characterization

The ^1^H NMR spectra of compounds (1a–1f) displayed characteristic C–H protons of the thiazol-2-imine ring appearing as singlets in the range of *δ* 6.87–7.12 ppm, and aromatic protons consistent with monosubstituted phenyl groups (AA′BB′C system) or *para*-disubstituted phenyl groups (AA′BB′ system) (Fig. S1, S5, S9, S13, S17 and S21). The ^13^C ATP NMR analysis also confirmed the formation of cyclized products with the characteristic thiazolyl methine carbon signal in the range *δ* 107.01–108.88 ppm (Fig. S2, S6, S10, S14, S18 and S22).

FT-IR spectroscopy revealed that compounds (1a–1f) have characteristic absorption bands for O–H, C=O, C=N, C–H (aromatic), and C–H (aliphatic) in their respective regions (Fig. S3, S7, S11, S15, S19 and S23). Notably, the absence of N–H stretches in the range 3200–3300 cm^−1^ across all spectra indicates loss of protons upon cyclization to form the imine. High-resolution mass spectrometry (HRMS) confirmed the expected molecular ion peaks for each compound (Fig. S4, S8, S12, S16, S20 and S24).

UV-vis spectra (recorded in DMSO) for compounds 1a–1f (Fig. S25), show an absorption band in the range 334–365 nm, which can tentatively be assigned to the allowed π–π* transition of the conjugated thiazole-2-imine/aromatic system. Note: mixing with n–π* cannot be excluded. These experimental features were validated using TD-DFT calculations, and the calculated absorption maxima (*λ*_cal_), excitation energies (*E*_ex_), oscillator strengths (*f*), and orbital contributions are summarized in Table S9 (gas phase) and Table S10 (C-PCM/DMSO).

### Crystal structures and DFT calculations

Single crystals suitable for X-ray diffraction were obtained for compounds 1b, 1c, and 1f by slow evaporation of DMSO solutions at ambient temperature. Data collection and refinement parameters are summarized in Table S1. The structures exhibit a U-conformation with respect to the –C(O)–N–C(S)< backbone typical of the acylthioureas (Fig. 2).

**Fig. 2:**
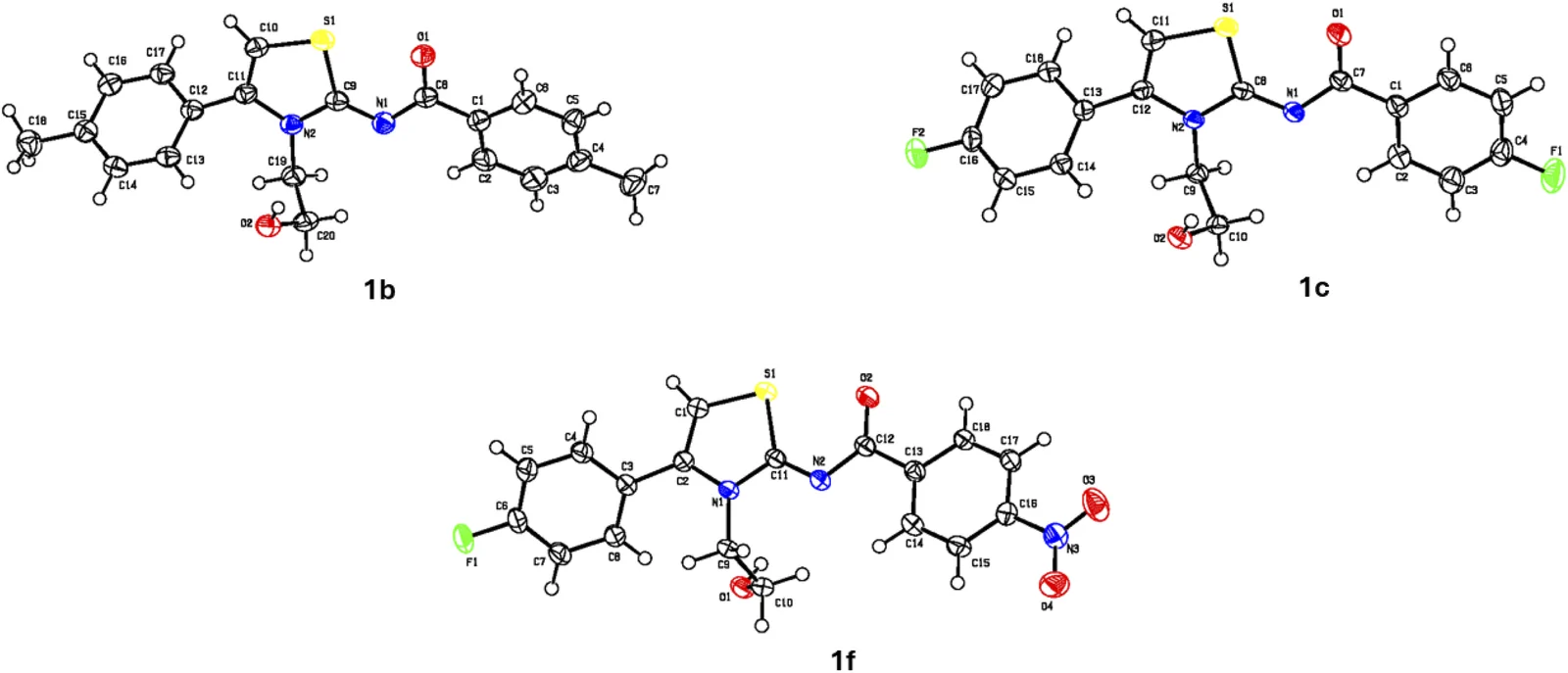
Crystal structures of (i) 1b, (ii) 1c, and (iii) 1f with displacement ellipsoids at 50%.

The crystal structures showcase the compounds' preference for the *Z*-configuration in the solid state. Crystal packing in these compounds is stabilized by a network of intermolecular hydrogen bonding, π–π stacking (offset parallel, tilted T-shaped), and alkyl C–H–π interactions (Fig. S26–S28).

Quantum chemical calculations were performed on structures 1a–1f. Geometry optimization was done in the gas phase using the following functional and basis set: B3LYP/6-311++G(d,p).^20^ The optimized geometries of 1b, 1c, and 1f had a match greater than 99.9% when overlaid onto their respective crystal structures along the acyl thiazol-2-imine backbone (Fig. S29). A comparison of bond lengths (Table S2) also showed that the optimized geometries closely matched the crystal structures.

The frontier molecular orbital (FMO) energies provide a qualitative measure of stability and chemical reactivity. Table 1 summarizes the global reactivity descriptors derived from the FMOs for compounds 1a–1f. The ionisation potential (*I*) shows the tendency to donate electrons, and the electron affinity (*A*) indicates the tendency to accept electrons.^21^ The HOMO–LUMO gap (*E*_g_) and the associated chemical hardness (*η*) are inversely related to reactivity. Global softness (*S*) increases as *E*_g_ decreases.^22^ The electronegativity (*χ*) and electrophilicity index (*ω*) describe the overall ability of a molecule to attract electrons and to behave as an electrophile in polar interactions.^23^ As shown in Table 1, the series displays comparable ionization potentials and electron affinities, with substituent-dependent shifts that modulate *E*_g_, *η*/*S*, and *ω*. The trends are consistent with the electronic effects of the para-substituents (where electron-withdrawing groups generally increase *χ*/*ω*, and electron-donating groups generally decrease them). These substituent-dependent variations in the calculated descriptors provide a quantitative basis for qualitatively assessing how structural modification alters the electronic character of this scaffold. Collectively, the calculated descriptors (Table 1) show that the thiazole-2-imine scaffold can be electronically tuned across the series, with possible implications for noncovalent interactions and structure–activity relationships.^24,25^ However, these descriptors alone do not predict bioactivity.

**Table 1:** Reactivity indices for compounds 1a–1f

Compound	*E*_HOMO_ (eV)	*E*_LUMO_ (eV)	*E*_g_ (eV)	*I* (eV)	*A* (eV)	*S* (eV)	*η* (eV)	*Χ* (eV)	*ω* (eV)
1a	−6.02	−1.62	4.41	6.02	1.62	0.454	2.20	3.82	3.31
1b	−5.93	−1.51	4.42	5.93	1.51	0.452	2.21	3.72	3.12
1c	−6.19	−1.76	4.43	6.19	1.76	0.451	2.22	3.97	3.57
1d	−5.77	−1.39	4.38	5.77	1.39	0.456	2.19	3.58	2.92
1e	−6.44	−2.89	3.55	6.44	2.89	0.564	1.77	4.66	6.13
1f	−6.53	−2.93	3.60	6.53	2.93	0.556	1.79	4.74	6.23

### Evaluating structural similarity to thiamine *via* flexophore descriptor analysis

A flexophore is a chemical descriptor used to assess molecular similarity by considering diverse conformer structures.^26,27^ In this study, flexophore descriptors were generated using DataWarrior^28^ to assess whether the synthesized thiazole-2-imine derivatives exhibit molecular and conformational similarity to thiamine. The similarity between thiamine (reference) and thiazole-2-imine derivatives (1a–f) is depicted in Fig. 3.

**Fig. 3:**
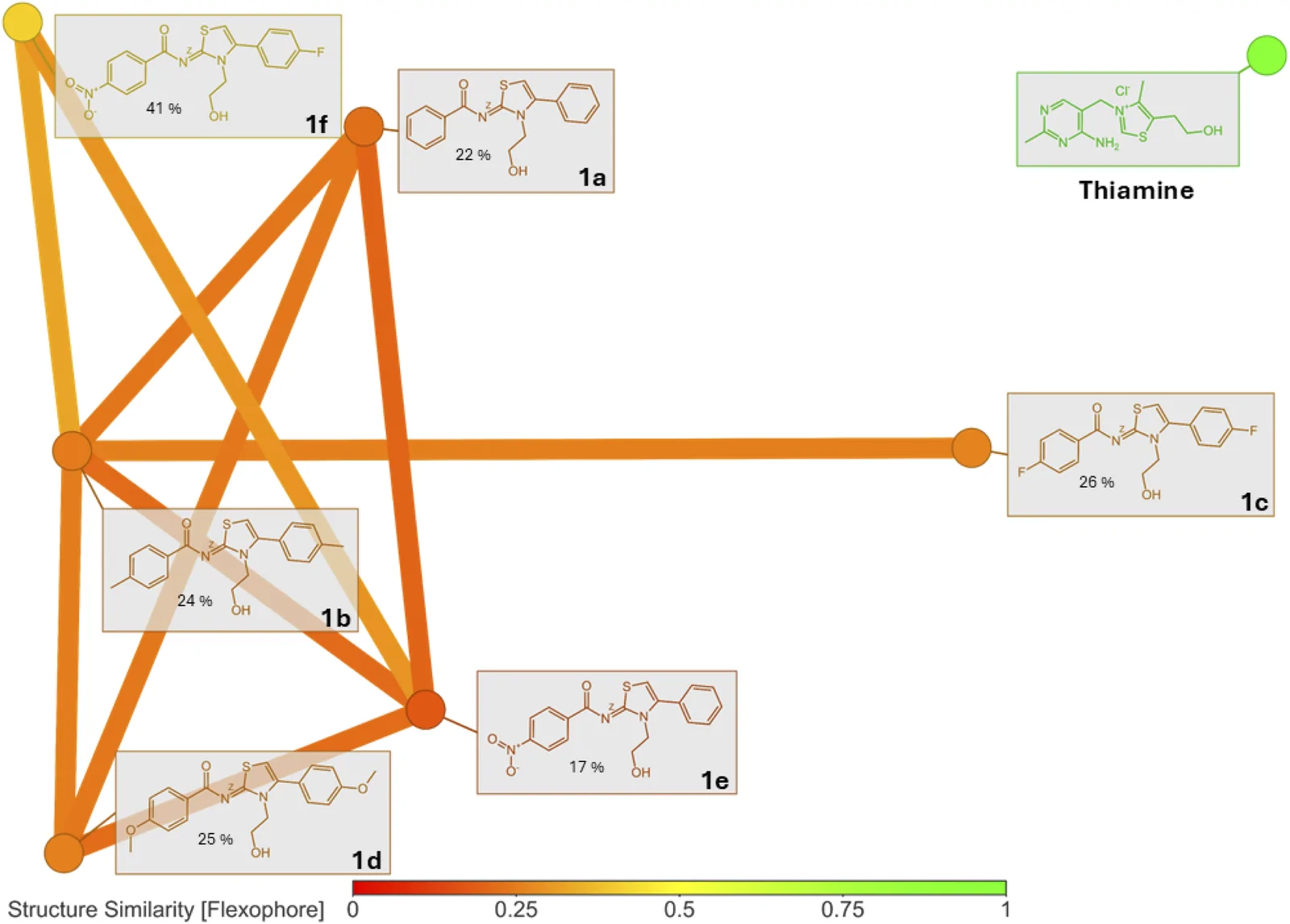
Similarity flexophore chart for compounds 1a–1f against thiamine (reference).

Compounds 1a–f exhibit low to moderate similarities with the reference thiamine, but have significant similarity among themselves (shown by color-based connecting lines). Compound 1f had the highest similarity score (41%), while others ranged from 17% to 26%.

### Molecular docking

Since flexophore analysis suggested partial retention of thiamine-related features, molecular docking was used to explore whether hypothetical pyrophosphorylated derivatives of the scaffold could be accommodated within selected human and microbial thiamine-dependent enzyme binding sites. The hypothetical pyrophosphorylated derivatives 1aPP–1fPP were computationally generated and used to investigate their possible docking modes within the cofactor-binding sites of *human pyruvate dehydrogenase* enzyme (PDB ID: 1NI4), *human transketolase* enzyme (PDB ID: 3MOS), and *M. tuberculosis transketolase* enzyme (PDB ID: 3RIM) using AutoDock Vina.^29,30^*Human pyruvate dehydrogenase* and *transketolase* were included to explore potential off-target models, in which favourable docking to these human enzymes was interpreted as a potential metabolic safety concern rather than as evidence of anti-infective significance. *M. tuberculosis transketolase* was explored for possible pathogen-relevant docking analysis.

Note: These derivatives were not synthesized or experimentally detected. This study also provides no evidence that compounds 1a–1f undergo pyrophosphorylation under biological conditions. Therefore, the docking results are hypothesis-generating and do not establish antimetabolite activity, enzyme inhibition, or intracellular formation of the pyrophosphorylated species. TPP's strong score on *human pyruvate dehydrogenase* (−9.6) provided a reference benchmark for interpreting the docking scores, as the hypothetical compounds (1aPP–1fPP) showed moderate to high docking scores (−6.6 to −8.3) (Table 2).

**Table 2:** Docking scores for hypothetical phosphorylated compounds 1aPP–1fPP against *human pyruvate dehydrogenase* enzyme (1NI4), *human transketolase* enzyme (3MOS), and *M. tuberculosis transketolase* enzyme (3RIM)

Compound (active form)	Docking score (kcal mol^−1^)	Docking score (kcal mol^−1^)	Docking score (kcal mol^−1^)
	1NI4	3MOS	3RIM
1aPP	−7.5	−7.9	−8.6^b^
1bPP	−6.6	−7.9	−7.5
1cPP	−7.5	−7.1	−8.8^a^
1dPP	−8.3	−7.2	−7.4
1ePP	−7.3	−7.5	−7.4^b^
1fPP	−7.3	−7.6	−7.7^a^
TPP	−9.6	−6.5	−9.2

a Top-scoring pose binds in a different orientation than the native ligand.

b Top-scoring pose binds differently, though a slightly lower-scoring pose matches the native ligand's binding mode.

Compound 1dPP exhibited the highest docking score (−8.3) to *human pyruvate dehydrogenase* (Fig. S30). Compounds (1aPP–1fPP) show better docking scores with *human transketolase* than TPP (−6.5), possibly due to favourable orientation or functional-group alignment. Compounds 1aPP and 1bPP had the best docking scores to *human transketolase*. The docking pose of 1aPP (Fig. 4) shows the pyrophosphate arm fixing the ligand in the binding pocket through interactions with the Ca^2+^ ion as well as amino acid residues Gly 156, ASN 185, ASP 155, LYS 244, and LYS 75, similar to the interactions of the native ligand TPP. However, there are additional hydrogen-bonding interactions with GLU 157, which are absent in the TPP docking pose. Additionally, there exist favourable alkyl-π interactions between the isobutyl chain of LEU 125 and the phenyl ring of 1aPP compared to the pyrimidine ring of TPP.

**Fig. 4:**
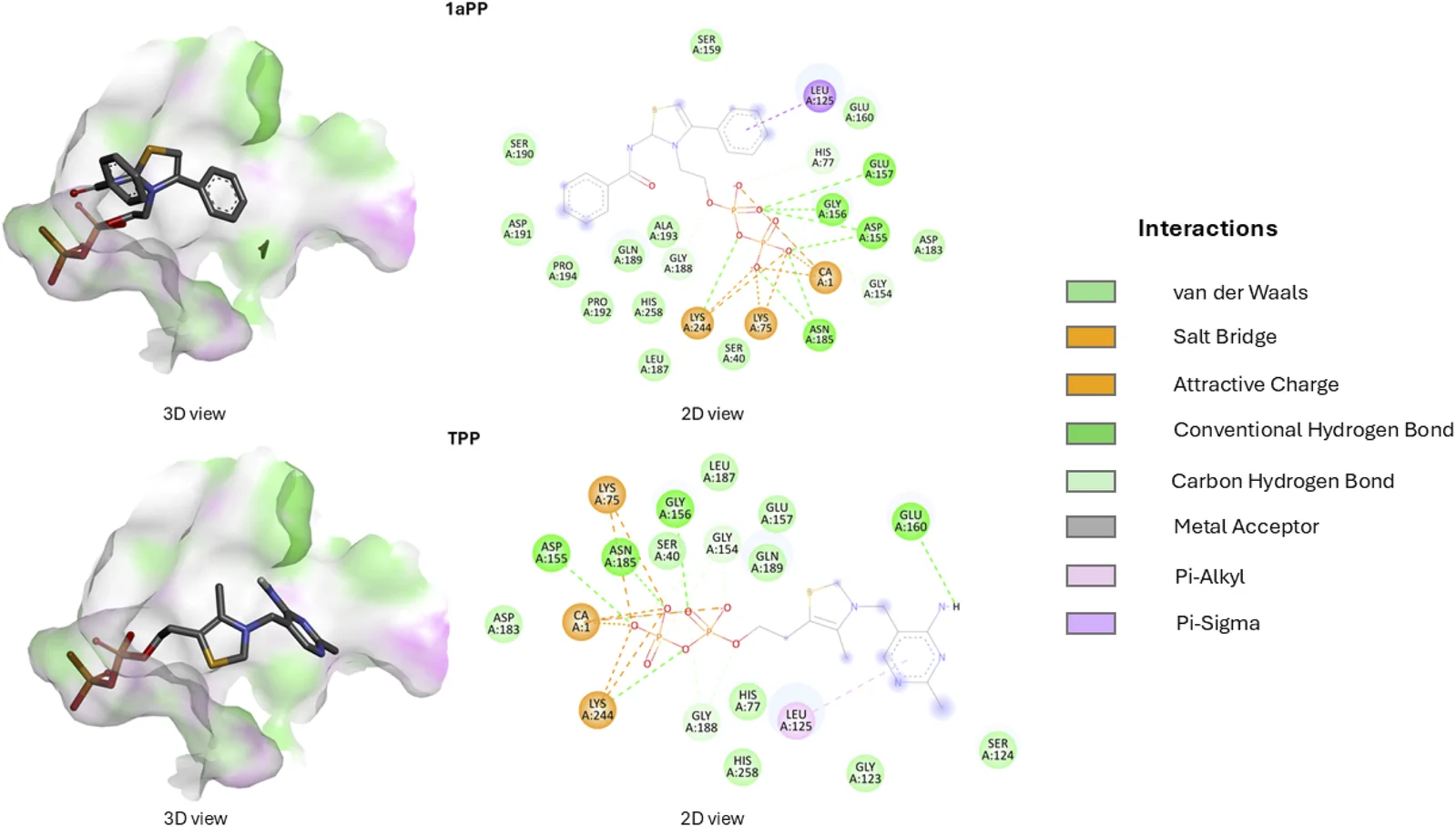
A comparison of the best docking poses for 1aPP and native TPP on human transketolase.

Docking against *Mycobacterium tuberculosis transketolase* showed that docking score and pose fidelity did not follow the same rank order. Native TPP gave the most favourable score (−9.2 kcal mol^−1^), while 1cPP (−8.8 kcal mol^−1^) and 1aPP (−8.6 kcal mol^−1^) were the closest-scoring derivatives. The top-ranked poses of 1bPP and 1dPP, bearing methyl and methoxy substituents, respectively, reproduced the TPP docking mode closely: their pyrophosphate tails were directed toward and interacted with the Mg^2+^ ion. By contrast, the best poses of 1aPP and 1ePP occupied the same cofactor cavity, but the Mg^2+^ ion interaction involved the aromatic region rather than the pyrophosphate tail. Nevertheless, slightly lower-ranked poses of both compounds adopted a TPP-like orientation. The fluorinated derivatives 1cPP and 1fPP also placed their aromatic fluoro-substituted regions toward Mg^2+^, and no alternative TPP-like poses were identified in the cavity. Collectively, these results indicate that hypothetical pyrophosphorylated derivatives of compounds 1a–1f could potentially be accommodated within selected human and microbial thiamine-dependent enzyme binding sites.

Compounds (1a–1f) were docked onto the *Mycobacterium tuberculosis InhA* enzyme (PDB ID: 8OTM), *Mycobacterium tuberculosis arabinosyltransferase* (PDB ID: 7BVF), *HIV-1 reverse transcriptase* (PDB ID: 1REV), and *human CCR5 chemokine* receptor (PDB ID: 4MBS) using AutoDock Vina.^30,31^ The results are presented in Table 3.

**Table 3:** Docking scores for compounds 1a–1f against four protein targets: 8OTM and 7BVF (TB-related), and 1REV and 4MBS (HIV-related)

Compound (inactive form)	Docking score (kcal mol^−1^)	Docking score (kcal mol^−1^)	Docking score (kcal mol^−1^)	Docking score (kcal mol^−1^)
	8MOT	1REV	7BVF	4MBS
1a	−6.3^a^	−8.4	−7.9	−7.5
1b	−6.3^a^	−8.6	−9.1	−8.6
1c	−6.7	−8.9	−8.2	−7.9^a^
1d	−6.3^a^	−8.5	−8.2	−7.7
1e	−6.5^a^	−8.4	−8.6	−7.9
1f	−5.8^a^	−8.7	−8.3	−8.1^a^
TB9 ligand	—	−9.2	—	—
VZI ligand	−11.7	—	—	—
Ethambutol	—	—	−5.1	—
Maraviroc	—	—	—	−10.9

a Poses that docked in the active pocket as the native ligand but were not the best poses (in terms of interactions).

This molecular docking investigation provided insights into the predicted docking scores and possible binding modes of the synthesized compounds across four protein targets: 8OTM and 7BVF (TB-related), and 1REV and 4MBS (HIV-related). Compounds 1b, 1c, 1d, 1e, and 1f exhibited favourable docking to 7BVF (−8.2 to −9.1 kcal mol^−1^), while moderate docking scores were observed with 8OTM (−5.8 to −6.7 kcal mol^−1^) (Table 3). The strong binding of *N*-((1-(3-hydroxy-4-phenoxybenzyl)-1*H*-1,2,3-triazol-4-yl)methyl)-2-oxo-2*H*-chromene-3-carboxamide (VZI ligand) to 8OTM (−11.7 kcal mol^−1^) supports its relevance as a reference TB-related docking target in this study. Compound 1b scored better than ethambutol in docking to 7BVF (−9.1 kcal mol^−1^), and its docking pose (Fig. 5) illustrates that it binds the same pocket as ethambutol, with additional favourable interactions. Compounds (1a–1f) showed favourable docking scores with 1REV (−8.4 to −8.9 kcal mol^−1^) and 4MBS (−7.5 to −8.6 kcal mol^−1^). Tivirapine (TB9 ligand) and Maraviroc docked strongly to 1REV (−9.2 kcal mol^−1^) and 4MBS (−10.9 kcal mol^−1^), respectively, providing a reference benchmark for interpreting the docking results.

**Fig. 5:**
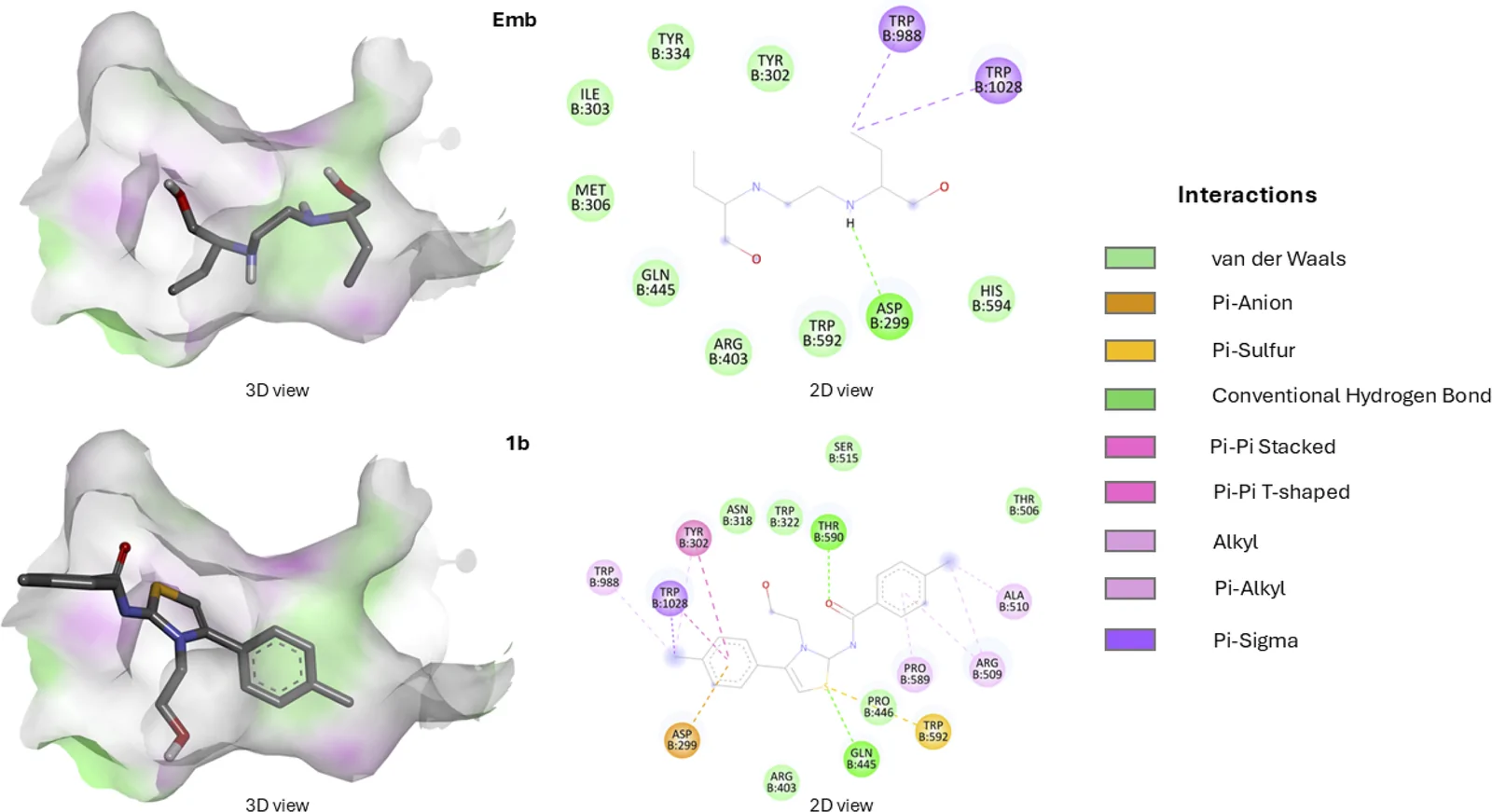
Best docking poses for compound 1b and ethambutol (Emb) on 7BVF protein.

### ADME/T study

AMDE/T properties of the compounds 1a–1f in this study are presented in Table 4. The parameters in this table examine the chemical properties of molecules: molar mass (mol MW), dipole moment (dipole), total solvent-accessible surface area (SASA), volume (volume), number of hydrogens (donorHB and acceptorHB), globularity descriptor (glob), and predicted polarizability (QPpolrz) values.

**Table 4:** *In silico* ADME/T property predictions of compounds 1a–1f^a^

Compound	QPlogKhsa	QPlogS	Human oral absorption (%)	QPlogBB	CNS	#metab	Ro5
1a	−0.701	−0.538	100.000	0.371	1	1	0
1b	−0.788	0.778	100.000	0.448	1	0	0
1c	−1.414	0.157	81.136	−0.035	−1	1	0
1d	−1.737	0.964	73.299	−0.155	−1	0	0
1e	−0.525	−1.704	100.000	0.316	1	0	0
1f	−1.650	0.382	74.455	−0.232	−1	0	0

a QPlogKhsa: predicted human serum albumin binding (−1.5 to 1.5); QplogS: predicted aqueous solubility (−6,5 to 0.5)%; human oral absorption: predicted human oral absorption (>80% = high and < 25% = poor); QplogBB: predicted brain/blood partition coefficient (−3 to 12.0); CNS: predicted central nervous system activity (−2 (inactive) to +2 (active)); #metab: number of likely metabolic reactions (1 to 8); Ro5: number of violations of Lipinski's rule of 5 (maximum = 4).

*In silico* ADME/T profiling revealed favourable predicted pharmacokinetic properties across the series. All compounds showed high predicted human oral absorption (≥74%), with 1a–1c and 1e achieving 100%. The predicted solubility (QPlogS) values ranged from −1.704 to 0.964. Most compounds fell within the recommended range of −6.5 to 0.5, whereas compounds 1b and 1d were outside this range, with values of 0.778 and 0.964, respectively. Serum albumin binding (QPlogKhsa) was within the recommended range (−1.737 to −0.525), suggesting moderate plasma protein interaction. Brain penetration (QPlogBB) was low to moderate, with central nervous system (CNS) activity predictions ranging from −1 to +1, suggesting limited CNS effects. Importantly, none of the compounds violated Lipinski's rule of five, and all the compounds showed low metabolic liability (#metab = 0–1), supporting their drug-likeness and oral bioavailability.

### *In vitro* anti-TB, anti-HIV, and cytotoxicity studies

Compounds 1a–1f were subsequently evaluated experimentally against *Mycobacterium tuberculosis* H37Rv (ATCC 27294), and moderate MIC_90_ values ranging from 125 to 250 µM were observed compared with the reference drug ethambutol (Table 5). Compounds 1b, 1c, 1e, and 1f are more active than 1a and 1d, consistently having MIC_90_ values of 125 µM.

**Table 5:** *In vitro* anti-TB activity against *Mycobacterium tuberculosis* H37Rv (ATCC 27294), anti-HIV activity against HIV-1 subtype B for compounds 1a–1f

Compound	Anti-*MTB* (µM)	Anti-HIV (µM)	Max inhibition (%)
	H_37_Rv MIC_90_	HIV-1 subtype B IC_50_	HIV-1 subtype B
1a	250	500	27
1b	125	190.5	51
1c	125	500	0
1d	250	500	16
1e	125	500	33
1f	125	75.86	66
Ethambutol	1.953	—	—
AZT	—	0.01327	99

*In vitro anti*-HIV activity of these compounds (Table 5) was investigated. Compounds 1b (IC_50_ of 190.50 µM) and 1f (IC_50_ of 75.86 µM) emerged as the most promising for further investigation (Fig. 6), while the other 6 derivatives exhibited weaker inhibition (IC_50_ = 500 µM). When compared to the reference drug, AZT, these compounds showed modest activity.

**Fig. 6:**
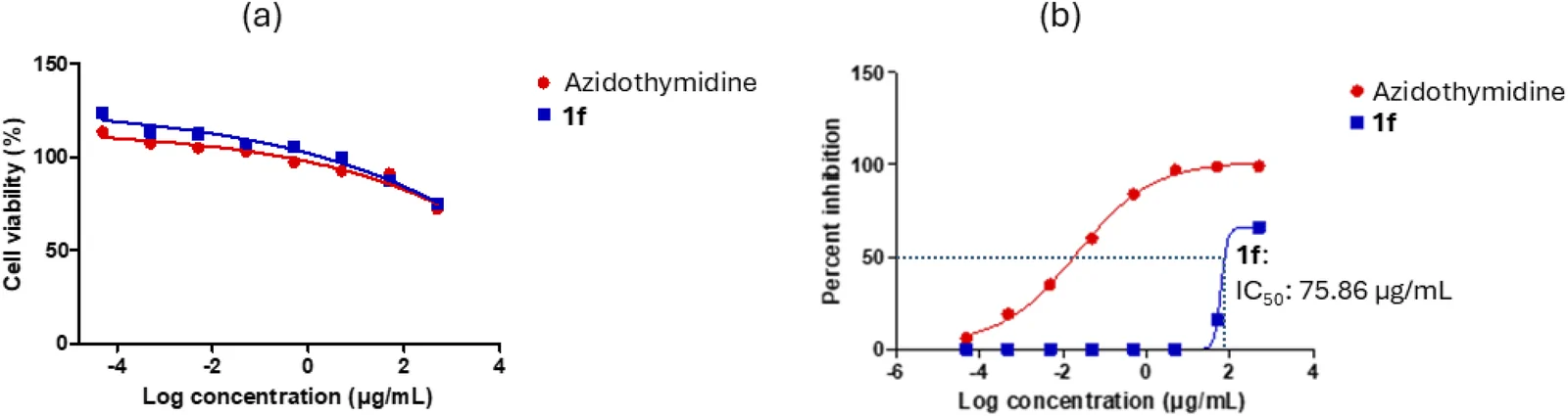
Cell viability and dose–response curves for compound 1f against HIV-1.

Cytotoxicity was assessed against the TZM-bl cell line. The CC_50_ values were used to calculate HIV-specific selectivity indices (SI = CC_50_/IC_50_) because SI requires cytotoxicity and antiviral activity to be evaluated in the same host cell line. Note: The reported TZM-bl cytotoxicity values (Table 6) are not used to infer general human cytotoxicity and are not associated with the anti-TB activity data.

**Table 6:** TZM-bl cytotoxicity (CC_50_) and HIV-1 subtype B selectivity index (SI) values for the tested compounds (1a–1f)^a^

Anti HIV assay							
Compound	1a	1b	1c	1d	1e	1f	AZT
Cytotoxicity CC_50_ (µM)	>500	>500	>500	>500	>500	>500	>300
Si^a^ (CC_50_/IC_50_)	1	2.625	1	1	1	6.591	226 074

a SI^a^: selectivity index.

Among the series, compound 1f exhibited the highest SI (although very low compared to the reference drug AZT), indicating a favourable balance between antiviral efficacy and host cell safety. Compound 1b followed with a lower SI. The remaining derivatives (1a, 1c, 1d, and 1e) had SI values close to 1, suggesting limited selectivity and a narrow therapeutic window. These findings reinforce compounds 1f and 1b as the most promising preliminary scaffolds, combining measurable but modest *in vitro* activity with a good HIV-based selectivity profile within the series.

## Conclusion

Six thiazol-2-imines were successfully synthesized *via* Hantzsch thiazole synthesis in excellent yields and characterized using various spectroscopic techniques as well as high-resolution mass spectrometry and single-crystal X-ray diffraction. The X-ray diffraction analysis revealed a preference for the *cis*/*Z* configuration in the solid state. The DFT calculations showed that the optimized geometries of the compounds closely matched the observed data in the crystal structures. Flexophore and molecular docking were used to support and provide hypothesis-generating context for this investigation. These analyses did not establish antimetabolite activity or specific molecular targets. The *in silico* ADME/T analysis showed that the majority of the synthesized compounds have acceptable physico-chemical properties. The synthesized compounds (1a–1f) achieved moderate *in vitro* activity (MIC_90_ = 125-250 µM) against the *Mtb H37Rv* strain when compared to the first-line drug ethambutol (MIC_90_ = 1.953 µM). The nitro/fluoro substituted derivative, 1f, displayed appreciable anti-HIV activity (IC_50_ of 75.86 µM) and a favourable selectivity index (6.59). This was followed by the methyl/methyl substituted derivative 1b, identifying both as preliminary scaffolds for further investigation and optimization.

## Experimental

### Materials and methods

All reagents and chemicals were purchased from Sigma Aldrich and used without further purification. Monosubstituted acylthioureas were prepared as reported in the literature.^17,18^ Melting points were measured using a Stuart SMP 30 apparatus and glass capillary tubes; they were recorded in degrees Celsius (°C) without correction. FT-IR spectra were obtained using a Bruker Tensor27 spectrometer in the range 4000-600 cm^−1^. UV-vis absorption spectra of compounds 1a–1f (in DMSO) were analysed at concentrations of 3.391 × 10^−5^, 3.405 × 10^−5^, 2.341 × 10^−5^, 3.330 × 10^−5^, 3.249 × 10^−5^, and 2.581 × 10^−5^ mol dm^−3^, respectively, using a 1.0 cm quartz cuvette on a Thermo Scientific Evolution 220 spectrophotometer in the 1000–200 nm range. NMR analysis was conducted on the Bruker 600 MHz spectrometer or the Bruker 400 MHz spectrometer using deuterated dimethyl sulfoxide (DMSO-*d*_6_) as solvent. MestReNova was used for spectral analysis; the coupling constants (*J*) are reported in Hertz (Hz), and the chemical shifts in parts per million (ppm) relative to DMSO-*d*_6_ [*δ* 2.50 ppm (^1^H) and *δ* 39.52 ppm (^13^C)]. The splitting patterns are reported as singlet (s), doublet (d), multiplet (m), triplet (t), and quartet (q). High-resolution mass spectra were recorded on a Waters Synapt G2 spectrometer with an electrospray ionization (ESI) probe.

Single-crystal X-ray diffraction data (SC-XRD) for the compounds (1b, 1c, and 1f) were collected on a Bruker Apex-II CCD diffractometer using Mo Kα radiation (*l* = 0.71073 A) at a temperature of 173(2) K. Intensities were integrated with SAINT+ (v6.02),^31^ and absorption corrections based on equivalent reflections were carried out using SADABS.^32^ The structures were solved using WinGx (v2014),^33^ refined using ShelXL,^34^ and OLEX2.^35^ All non-hydrogen atoms were refined anisotropically, while all hydrogen atoms were located geometrically and refined using a riding model. Data collection and refinement parameters are summarized in Table S1. CCDC numbers for (*Z*)-*N*-(3-(2-hydroxyethyl)-4-(*p*-tolyl)thiazol-2(3*H*)-ylidene)-4-methylbenzamide (1b), (*Z*)-4-fluoro-*N*-(4-(4-fluorophenyl)-3-(2-hydroxyethyl)thiazol-2(3*H*)-ylidene)benzamide (1c), and (*Z*)-*N*-(4-(4-fluorophenyl)-3-(2-hydroxyethyl)thiazol-2(3*H*)-ylidene)-4-nitrobenzamide (1f) are 2549158, 2549159, and 2549160 respectively. Diagrams and publication materials were generated using PLATON^36^ and MERCURY.^37^

#### General synthesis of thiazole-2-imine derivatives (1a–f)

Respective monosubstituted acylthioureas (0.5 mmol) were dissolved in acetone (10 mL) in a 50 mL round-bottomed flask. Corresponding equimolar amounts of a *para*-substituted phenacyl bromide (phenacyl bromide, 4-methyl-phenacyl bromide, 4-fluoro-phenacyl bromide, or 4-nitro-phenacyl bromide) were added, and the reactions were stirred at ambient temperature for 12 h. After the reactions were complete, as indicated by TLC, the resulting precipitates were filtered, washed with distilled water and a minimal amount of methanol, affording the products in very good yields and acceptable purity. The products were characterized using ^1^H and ^13^C NMR spectroscopy, FT-IR spectroscopy, HRMS, UV-vis spectrophotometry, and single-crystal X-ray diffraction.

##### (*Z*)-*N*-(3-(2-Hydroxyethyl)-4-phenylthiazol-2(3*H*)-ylidene)benzamide (1a)

Yield: 82%; mp (172.2–172.6 °C); UV (DMSO), *λ*/nm (*ε*/dm^3^ mol^−1^ cm^−1^): 336 (24 131); FT-IR (ATR): *v* = 3303–3354 (w, O–H), 3062–3085 (w, aromatic C–H), 2881–2935 (w, aliphatic C–H), 1593 (m), 1549 (m C=O), 1477 (s, C=N), 1371 (s), 1059 (m), 908 (m), 712 (s) cm^−1^; 1H NMR (600 MHz, DMSO-*d*_*6*_): *δ*_H_ 8.28–8.17 (m, 2H), 7.65–7.58 (m, 2H), 7.56–7.53 (m, 4H), 7.52–7.48 (m, 2H), 7.00 (s, 1H), 4.99 (d, *J* = 5.3 Hz, 1H), 4.26 (t, *J* = 6.1 Hz, 2H), 3.76 (q, *J* = 5.4 Hz, 2H) ppm; ^13^C NMR (101 MHz, DMSO-*d*_*6*_): *δ*_C_ 172.97, 168.35, 140.05, 137.35, 131.98, 131.08, 130.37, 129.90, 129.19, 129.17, 128.70, 107.82, 58.06, 49.64 ppm; HRMS (ESI^+^) *m*/*z* calculated for C_18_H_17_N_2_O_2_S [(M + H)]^+^ 325.1011, found: 325.1000.

##### (*Z*)-*N*-(3-(2-Hydroxyethyl)-4-(*p*-tolyl)thiazol-2(3*H*)-ylidene)-4-methylbenzamide (1b)

Yield: 83%; mp (167.0–167.5 °C); UV (DMSO), *λ*/nm (*ε*/dm^3^ mol^−1^ cm^−1^): 336 (21 084); FT-IR (ATR): *v* = 3344 (w, O–H), 3001–3037 (w, aromatic C–H), 2885–2950 (w, aliphatic C–H), 1593 (m), 1545 (m C=O), 1477 (s, C=N), 1360 (s), 1059 (m), 906 (m), 756 (s) cm^−1^; 1H NMR (600 MHz, DMSO-*d*_*6*_) *δ*_H_ 8.13–8.08 (m, 2H), 7.50–7.45 (m, 2H), 7.34 (d, *J* = 7.6 Hz, 2H), 7.30 (d, *J* = 7.8 Hz, 2H), 6.91 (s, 1H), 4.23 (t, *J* = 6.2 Hz, 2H), 3.73 (t, *J* = 6.2 Hz, 2H), 2.39 (s, 3H), 2.38 (s, 3H) ppm; ^13^C NMR (101 MHz, DMSO-*d*_6_) *δ*_C_ 172.91, 168.15, 141.89, 140.00, 139.50, 134.72, 130.22, 129.72, 129.28, 129.26, 128.17, 107.37, 58.08, 49.52, 21.61, 21.35 ppm; HRMS (ESI^+^) *m*/*z* calculated for C_20_H_21_N_2_O_2_S [(M + H)]^+^ 353.1324, found: 353.1314.

##### (*Z*)-4-Fluoro-*N*-(4-(4-fluorophenyl)-3-(2-hydroxyethyl)thiazol-2(3*H*)-ylidene)benzamide (1c)

Yield: 90%; mp (233.8–234.5 °C); UV (DMSO), *λ*/nm (*ε*/dm^3^ mol^−1^ cm^−1^): 334 (22 604); FT-IR (ATR): *v* = 3340 (w, O–H), 3070 (w, aromatic C–H), 2883–2952 (w, aliphatic C–H), 1601 (m), 1554 (m C=O), 1506 (s), 1485 (s, C=N), 1423 (s), 1363 (s), 1057 (m), 852 (m), 769 (m) cm^−1^; 1H NMR (600 MHz, DMSO-*d*_6_) *δ*_H_ 8.30–8.24 (m, 2H), 7.71–7.64 (m, 2H), 7.43–7.35 (m, 2H), 7.34–7.29 (m, 2H), 7.02 (s, 1H), 4.98 (t, *J* = 5.5 Hz, 1H), 4.22 (t, *J* = 6.0 Hz, 2H), 3.75 (q, *J* = 5.8 Hz, 2H) ppm; 13C NMR (101 MHz, DMSO-*d*_*6*_) *δ*_C_ 171.92, 168.27, 165.98, 164.41, 163.51, 161.96, 139.08, 133.93, 133.90, 132.89, 132.81, 131.79, 131.70, 127.46, 127.43, 116.25, 116.04, 115.71, 115.49, 108.13, 58.04, 49.66 ppm; HRMS (ESI^+^) *m*/*z* calculated for C_18_H_15_N_2_O_2_SF_2_ [(M + H)]^+^ 361.0822, found: 361.0815.

##### (*Z*)-*N*-(3-(2-Hydroxyethyl)-4-(4-methoxyphenyl)thiazol-2(3*H*)-ylidene)-4-methoxybenzamide (1d)

Yield: 91%; mp (152.0–152.5 °C); UV (DMSO), *λ*/nm (*ε*/dm^3^ mol^−1^ cm^−1^): 338 (26 821); FT-IR (ATR): *v* = 3330 (w, O–H), 3031 (w, aromatic C–H), 2947 (w, aliphatic C–H), 1604 (m), 1510 (m C=O), 1361 (s, C=N), 1253 (s), 1016 (m), 833 (m), 773 (m) cm^−1^; 1H NMR (600 MHz, DMSO-*d*_6_) *δ*_H_ 8.17 (d, *J* = 8.4 Hz, 2H), 7.56–7.49 (m, 2H), 7.11–7.05 (m, 2H), 7.04–6.99 (m, 2H), 6.87 (s, 1H), 4.21 (t, *J* = 6.2 Hz, 2H), 3.83 (s, 6H), 3.75 (t, *J* = 6.2 Hz, 2H) ppm; 13C NMR (101 MHz, DMSO-*d*_6_) *δ*_C_ 172.47, 167.83, 162.48, 160.50, 139.78, 131.79, 131.12, 129.88, 123.12, 114.58, 113.94, 107.01, 58.10, 55.80, 55.78, 49.49 ppm; HRMS (ESI^+^) *m*/*z* calculated for C_20_H_21_N_2_O_4_S [(M + H)]^+^ 385.1222, found: 385.1212.

##### (*Z*)-*N*-(3-(2-Hydroxyethyl)-4-phenylthiazol-2(3*H*)-ylidene)-4-nitrobenzamide (1e)

Yield: 95%; mp (172.1–172.9 °C); UV (DMSO), *λ*/nm (*ε*/dm^3^ mol^−1^ cm^−1^): 365 (11 443); FT-IR (ATR): *v* = 3406 (w, O–H), 3097 (w, aromatic C–H), 2962 (w, aliphatic C–H), 1556 (m C=O), 1471 (s, C=N), 1340 (s), 1061 (m), 850 (m), 721 (m) cm^−1^; 1H NMR (600 MHz, DMSO-*d*_6_) *δ*_H_ 8.43 (d, *J* = 8.9 Hz, 2H), 8.34 (d, *J* = 8.8 Hz, 2H), 7.65–7.60 (m, 2H), 7.57–7.52 (m, 3H), 7.11 (s, 1H), 5.00 (t, *J* = 5.6 Hz, 1H), 4.31 (t, *J* = 6.1 Hz, 2H), 3.75 (q, *J* = 5.9 Hz, 2H) ppm; 13C NMR (101 MHz, DMSO-*d*_*6*_) *δ*_C_ 170.98, 168.68, 149.67, 142.97, 140.49, 130.85, 130.41, 130.36, 130.03, 129.21, 124.00, 108.65, 58.08, 49.82 ppm; HRMS (ESI^+^) *m*/*z* calculated for C_18_H_16_N_3_O_4_S [(M + H)]^+^ 370.0862, found: 370.0857.

##### (*Z*)-*N*-(4-(4-Fluorophenyl)-3-(2-hydroxyethyl)thiazol-2(3*H*)-ylidene)-4-nitrobenzamide (1f)

Yield: 92%; mp (230.4–231.2 °C); UV (DMSO), *λ*/nm (*ε*/dm^3^ mol^−1^ cm^−1^): 365 (8474); FT-IR (ATR): *v* = 3379 (w, O–H), 3076 (w, aromatic C–H), 2943 (w, aliphatic C–H), 1556 (m C=O), 1425 (s, C=N), 1342 (s), 1053 (m), 833 (m), 721 (s) cm^−1^; 1H NMR (600 MHz, DMSO-*d*_6_) *δ*_H_ 8.43 (d, *J* = 8.8 Hz, 2H), 8.34 (d, *J* = 8.8 Hz, 2H), 7.72–7.65 (m, 2H), 7.40 (t, *J* = 8.9 Hz, 2H), 7.12 (s, 1H), 4.98 (t, *J* = 5.5 Hz, 1H), 4.27 (t, *J* = 5.9 Hz, 2H), 3.76 (q, *J* = 5.7 Hz, 2H) ppm; 13C NMR (101 MHz, DMSO-*d*_6_) *δ*_C_ 171.00, 168.58, 164.06, 162.42, 149.69, 142.95, 139.49, 132.96, 132.90, 130.36, 127.28, 127.26, 124.00, 116.26, 116.12, 108.88, 58.09, 49.86 ppm; HRMS (ESI^+^) *m*/*z* calculated for C_18_H_15_N_3_O_4_SF [(M + H)]^+^ 388.0767, found: 388.0763.

### Computational details

#### DFT study

Quantum chemical calculations were performed on compounds 1a–1f. Geometry optimization of the molecules was carried out in the gas phase using density functional theory (DFT) at the Becke-3-Lee-Yang-Parr/6-311++G(d,p) level of theory.^38–40^ Subsequent frequency calculations were then performed on the optimized geometries to ensure that the geometries correspond to minima. Tables S3–S8 present the *xyz* coordinates of the optimized geometries. Molecular reactivity descriptors were calculated (eqn (1)–(8)) from the frontier molecular orbital (FMO) energies (*E*_HOMO_ and *E*_LUMO_).^41,42^ The FMO and electrostatic potential (ESP) maps were visualised to identify reactive sites.^43,44^

1 *I* = −*E*_HOMO_

2 *A* = −*E*_LUMO_

3 *E*_g_ = *E*_LUMO_ − *E*_HOMO_

4
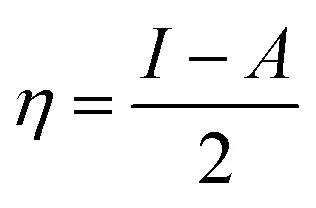
5
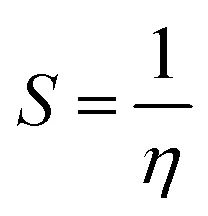
6
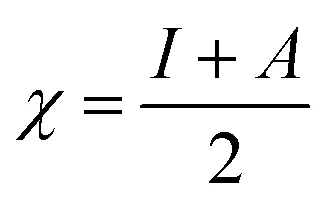
7
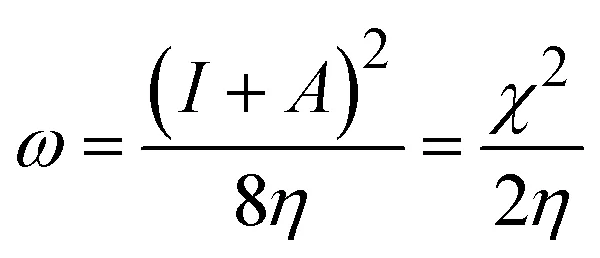


Electronic transitions of the compounds were investigated using time-dependent DFT (TD-DFT). The 6-311++G(d,p) level of theory was used for 1a–1d. It has been noted that typical DFT calculations do not adequately account for charge-transfer states and intermolecular solvent–solute interactions in nitro-substituted compounds in DMSO (generally overestimation of the absorption wavelength).^45,46^ Therefore, the Coulomb-attenuated method (CAM-B3LYP) at the same level of theory was used for 1e and 1f because of the nitro substituents. The experimental UV-vis spectra were measured in DMSO; as a result, electronic absorption properties were also calculated in DMSO using the conductor-like polarizable continuum model (C-PCM), which accounts for solute–solvent electrostatic interactions.^47^

#### Molecular docking

Molecular docking was carried out using AutoDock Vina 1.2.0.^29,30^ Crystal structures of *human pyruvate dehydrogenase* (PDB ID: 1NI4), *human transketolase* (PDB ID: 3MOS), *Mycobacterium tuberculosis* InhA (PDB ID: 8OTM), *Mycobacterium tuberculosis* arabinosyltransferase (PDB ID: 7BVF), HIV-1 reverse transcriptase (PDB ID: 1REV), and human CCR5 chemokine receptor (PDB ID: 4MBS) were retrieved from the Protein Data Bank. Protein preparation was done by removing crystallographic water molecules and non-relevant co-crystallized ligands, adding polar hydrogens, and assigning Gasteiger charges. The prepared receptors were saved in PDBQT format. Ligand structures (compounds 1a–1f, the pyrophosphorylated derivatives 1aPP–1fPP, and reference ligands) were minimized and converted to PDBQT format with rotatable bonds defined. Docking search spaces were defined by centering the grid box on the binding site of the co-crystallized ligand. Grid box dimensions were set to encompass the binding pocket and allow for ligand flexibility. Docking calculations were run with the number of runs set to 8. For each ligand the top-ranked poses were retained. Also, poses that were not top-ranked but docked in the same binding site as the native ligands were retained. To computationally check the docking protocol, the native ligands were re-docked into their corresponding receptors, and the predicted poses were compared to the crystallographic binding mode. Binding affinities (kcal mol^−1^) and docking poses were obtained from Vina output files. Protein–ligand interactions were inspected, and figures were generated using Biovia Discovery Studio Visualizer v21.^48^

### Biology

#### Antimycobacterial evaluation

Antimycobacterial activity was evaluated against *M. tuberculosis H37Rv* (ATCC 27294) using a resazurin microtiter assay as previously described.^49,50^ The strain was cultured aerobically in Middlebrook 7H9 broth supplemented with 0.1% glycerol and 10% OADC at 37 °C to OD_600_ = 1 (≈3 × 10^8^ bacilli per mL). Compounds 1a–1f were tested in triplicate by two-fold serial dilution in 96-well plates, sealed, and incubated at 37 °C for 7 days. Growth was assessed by the colour change (blue to pink) of resazurin. The MIC was defined as the lowest compound concentration required to prevent colour change.

#### MTT cytotoxicity evaluation

Cytotoxicity was assessed in TZM-bl (HeLa-derived) cells using an MTT assay as reported.^50,51^ Cells (25 000 per well) were seeded in 96-well plates in 150 µL DMEM containing DEAE–dextran (44 µL/10 mL) and incubated overnight (37 °C, 5% CO_2_). Serial dilutions, prepared from the stock solutions (2.5 mg mL^−1^), were added after the medium was removed. MTT reagent (20 µL of 5 mg mL^−1^ MTT in 0.1 M PBS + 100 µL fresh complete culture medium) was then added, and plates were incubated for 4 h (37 °C, 5% CO_2_). Absorbance was read at 450 nm (PerkinElmer microplate reader), and CC_50_ values were obtained by nonlinear regression (dose–response; concentration *vs.* % viability) in GraphPad Prism v5.01.

### Anti-HIV evaluation

#### Maintenance of cell lines

TZM-bl cell lines (NIH AIDS Research and Reference Reagents Programme) were cultured as a monolayer using Dulbecco's Modified Eagle Medium (DMEM) (Thermo Fisher Scientific, Waltham, MA, USA). In sterile 75 cm^2^ culture flasks, supplemented with 10% foetal bovine serum (FBS; heat-inactivated and gamma-irradiated) (LTC Biosciences, Gainesville, FL, USA), 25 mM of HEPES (Thermo Fisher Scientific, Waltham, MA, USA), and 50 µL mL^−1^ gentamicin (Thermo Fisher Scientific, Waltham, MA, USA). The TZM-bl cell line, a HeLa cell line clone, is engineered to express CD4 and CCR5, enabling HIV-1 infection and firefly luciferase expression regulated by the HIV-1 long terminal repeat (LTR).^52^

#### Antiviral assay

Anti-HIV activity was determined in TZM-bl cells using a luciferase reporter assay as previously reported.^50,51^ AZT (positive control) and test compounds (1a–1f) were added to 96-well plates and serially diluted (3-fold) in DMEM supplemented with 10% FBS, 25 mM HEPES, and 50 µL mL^−1^ of gentamicin (Thermo Fisher Scientific, Waltham, MA, USA). TZM-bl cells (10 000 cells per well) were infected with HIV-1 NL4.3 (subtype B; 50 µL) and incubated at 37 °C, 5% CO_2_, 95% humidity in the presence of DEAE–dextran (37.5 µg mL^−1^). Infected (virus control) and uninfected (cell control) wells were included. After 48 h, the culture medium was removed, and Bright-Glo™ luciferase reagent was added to quantify infection. The lysates were transferred to black 96-well plates, and luminescence was measured immediately using a Victor Nivo microplate reader (PerkinElmer).8
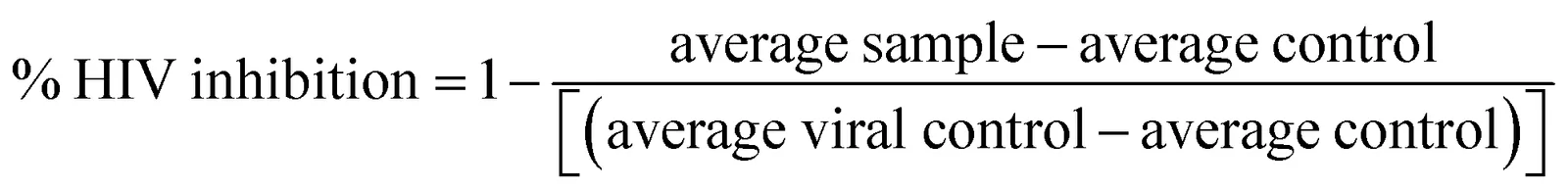


IC_50_ values were determined from dose–response curves (log concentration *vs.* % HIV inhibition) (Fig. 6).

## Author contributions

Edmore F. Kangara: experimental work, data curation, investigation, and writing – original draft, writing – reviewing and editing. Thishana Singh: conceptualization, project administration and writing – review and editing. Phindile Khoza: conceptualization, project administration, and writing – review and editing. Darian Naidu: experimental investigation and writing – review. Nompumelelo P. Mkwananzi: supervision, conceptualization, data curation, and writing – review and editing. Sibusiso Senzani: supervision, experimental work, data curation, writing – review and editing. Oluwatoba E. Oyeneyin: investigation, data curation, writing – review and editing. Manuel A. Fernandes: investigation, experimental work, data curation and writing – review and editing. Werner Van Zyl: conceptualization, project administration, supervision, investigation, and writing – review and editing. Matshawandile Tukulula: conceptualization, project administration, supervision, investigation, and writing – review and editing.

## Conflicts of interest

There are no conflicts to declare.

## Supplementary Material

RA-OLF-D6RA07024H-s001

## Data Availability

CCDC 2549158–2549160 contain the supplementary crystallographic data for this paper.^53*a*–*c*^ The NMR, FT-IR, HRMS, and SC-XRD data is available in the supplementary information (SI). Supplementary information is available. See DOI: https://doi.org/10.1039/d6ra07024h.
