# Supplementary material for: Synthesis, characterization, X-ray diffraction, in silico studies and in vitro biological evaluation of acylthiourea-derived thiazole-2-imines
Source: RSC Adv. 2026 Oct 8;16(46):60872–85. doi: 10.1039/d6ra07024h (PMC13646916; doi:10.1039/d6ra07024h)
Supplement: Supplementary file 1 [file RA-016-D6RA07024H-s001.pdf]

Supplementary Information (SI) for RSC Advances.
This journal is © The Royal Society of Chemistry

Supporting Information

Synthesis, characterization, X-ray diffraction, in silico studies and in vitro biological evaluation of acylthiourea-derived thiazole-2-imines.

Edmore F. Kangara [a,b], Werner Van Zyl [b], Thishana Singh [b], Phindile Khoza [b], Darian Naidu [c], Nompumelelo P. Mkwananzi [c], Sibusiso Senzani [d], Oluwatoba E. Oyeneyin [e], Manuel A. Fernandes [a] and Matshawandile Tukulula [b*].*

[a]Molecular Sciences Institute, School of Chemistry, University of the Witwatersrand, Private Bag X3, Johannesburg, 2050, South Africa.

[b]Discipline of Chemistry, School of Agriculture and Engineering, University of KwaZulu-Natal, Private Bag X54001, Westville, Durban, 4000, South Africa.

[c]HIV Pathogenesis Programme, Doris Duke Medical Research Institute, School of Laboratory Medicine and Medical Sciences, College of Health Sciences, University of KwaZulu-Natal, Durban, 4001, South Africa.

[d]School of Laboratory Medicine and Medical Science, College of Health Sciences, University of KwaZulu-Natal, Medical School Campus, Durban 4001, South Africa.

[e]Department of Chemical and Physical Sciences, Walter Sisulu University (WSU), Private Bag X01, Mthatha, Eastern Cape, 4099, South Africa.

Characterisation spectral data

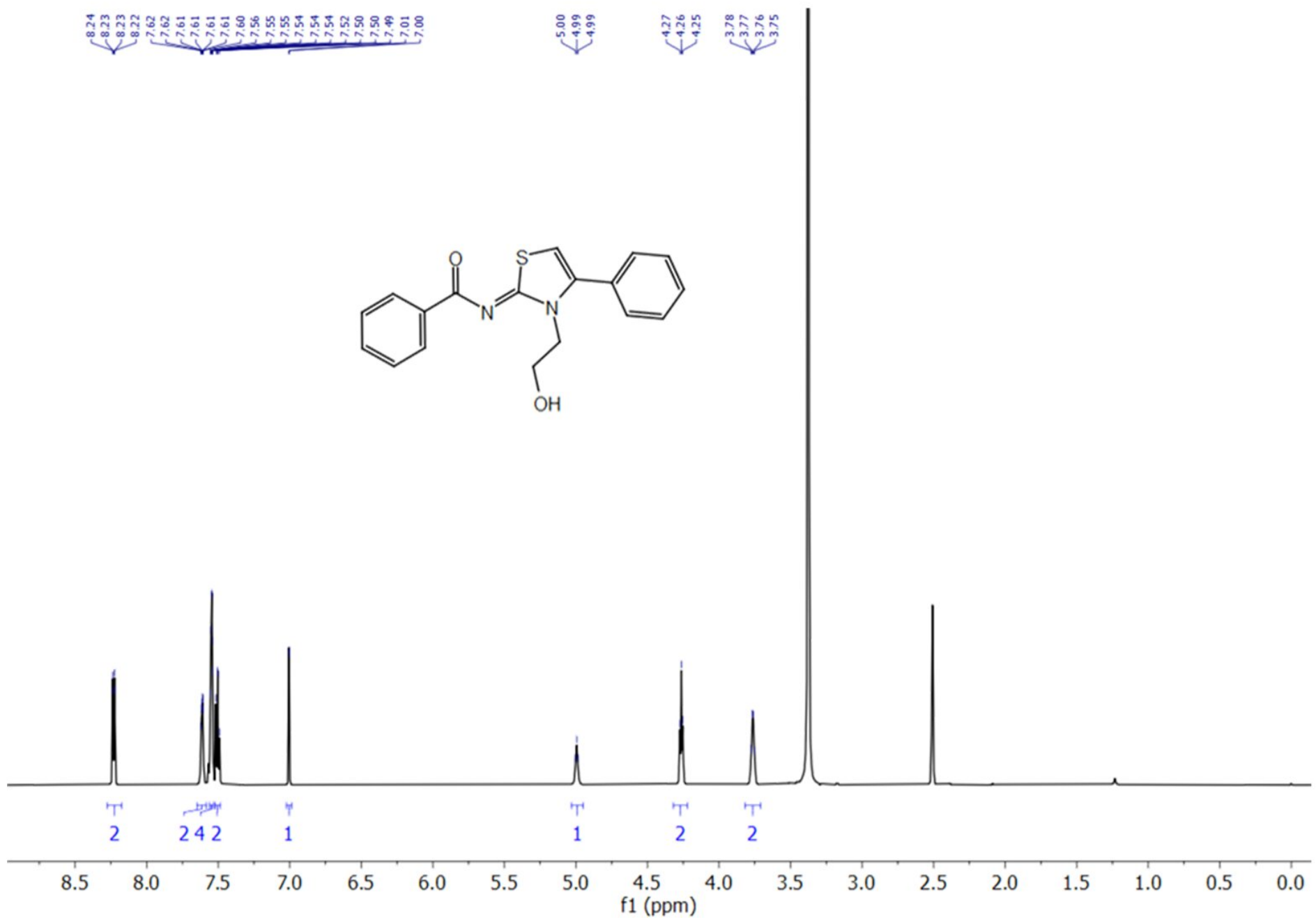

*Figure S 1: ^{1}H-NMR spectrum for compound **1a** in DMSO-d_6*

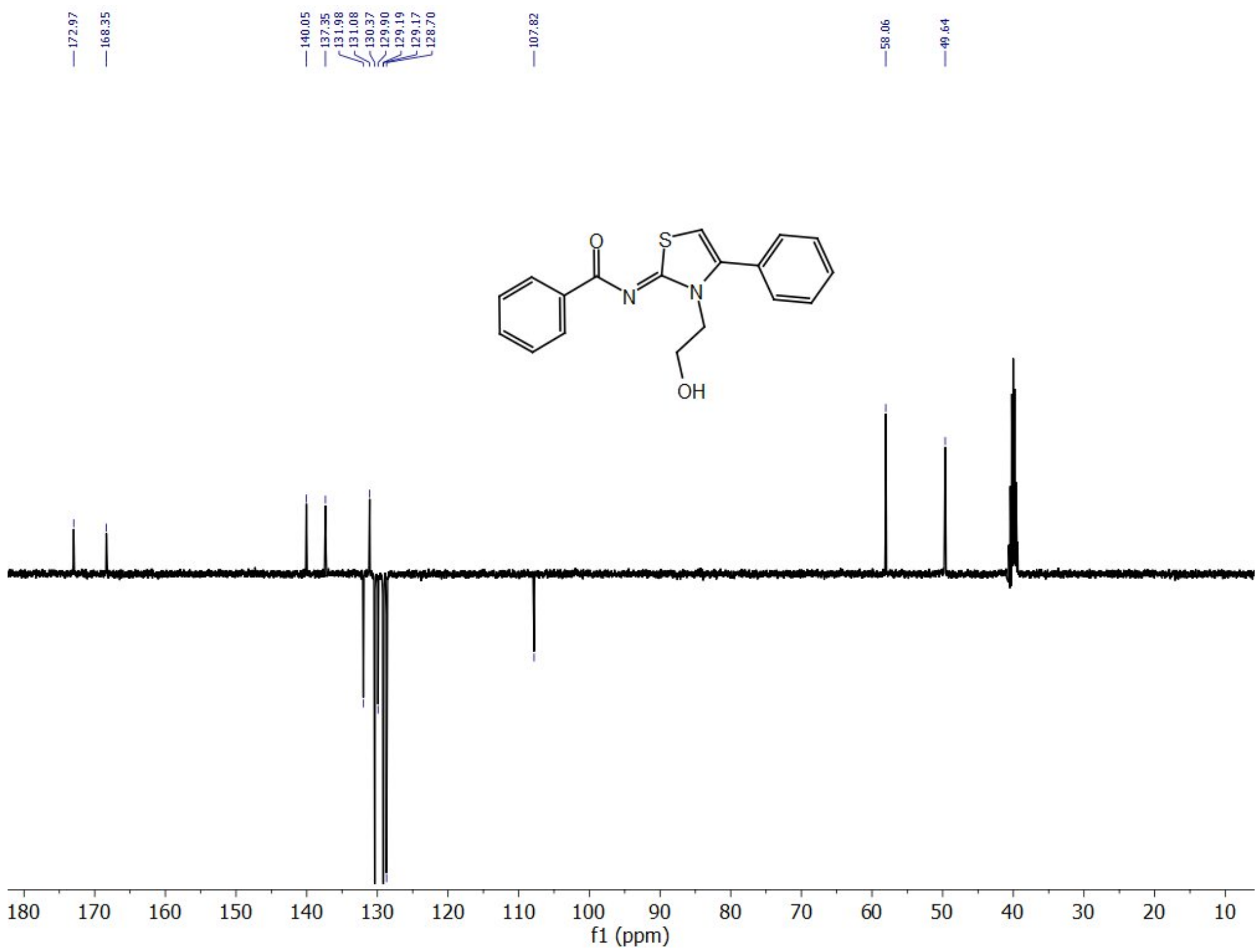

*Figure S 2: ^{13}C-NMR APT spectrum for compound **1a** in DMSO-d_6*

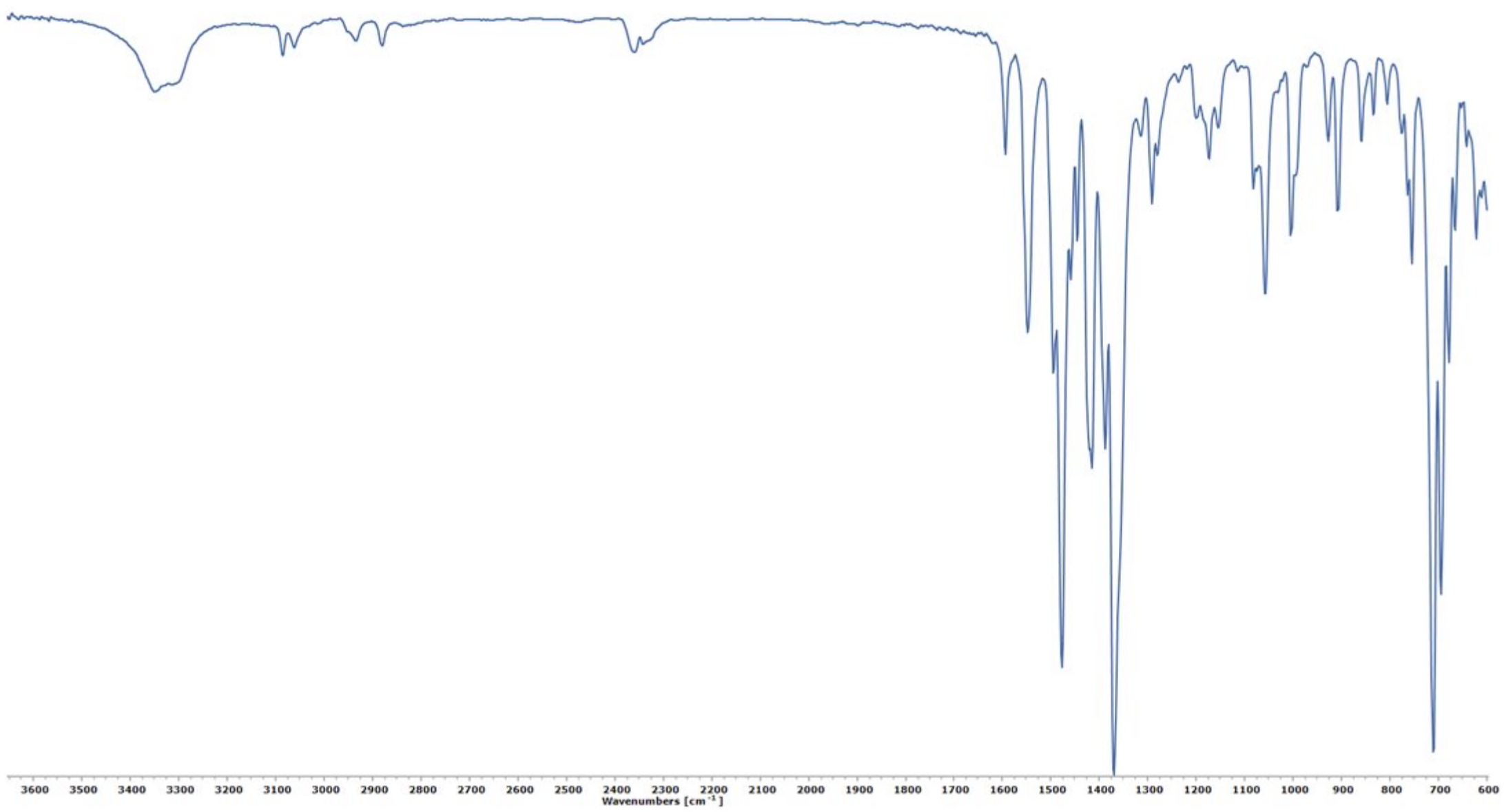

*Figure S 3: FTIR spectrum for compound **1a***

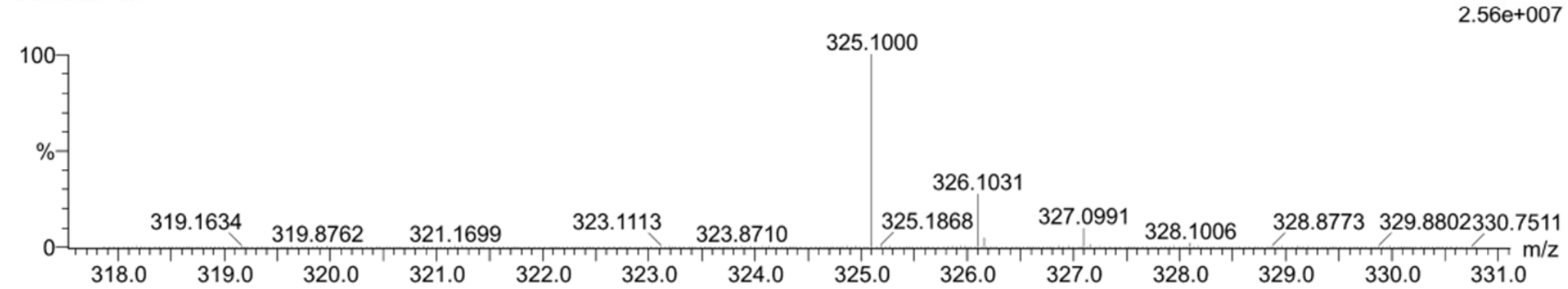

Elemental Composition Report — **Page 1**

Single Mass Analysis
Tolerance = 5.0 PPM / DBE: min = -5.0, max = 50.0
Element prediction: Off
Number of isotope peaks used for i-FIT = 3

Monoisotopic Mass, Even Electron Ions
339 formula(e) evaluated with 1 results within limits (all results (up to 1000) for each mass)
Elements Used:
C: 10-50 H: 0-20 N: 0-5 O: 0-5 S: 0-5
UNCY 40 (0.359) Cm (2:230)
TOF MS ES+

Minimum:				-5.0				
Maximum:		2.0	5.0	50.0				
Mass	Calc. Mass	mDa	PPM	DBE	i-FIT	Norm	Conf(%)	Formula
325.1000	325.1011	-1.1	-3.4	11.5	1277.0	n/a	n/a	C18 H17 N2 O2 S

*Figure S 4: HRMS spectrum for compound **1a** in methanol.*

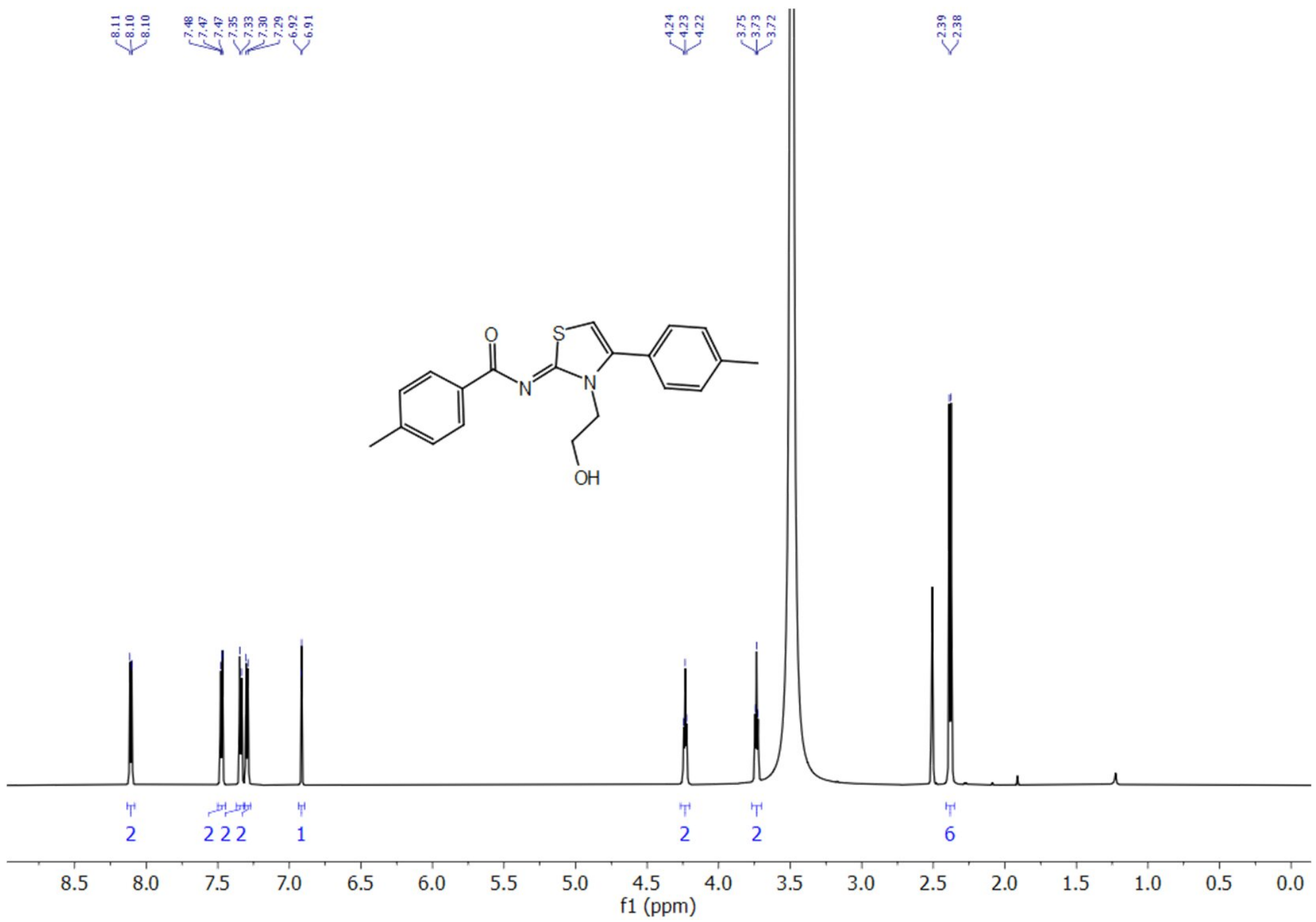

Figure S 5: ^{1}H-NMR spectrum for compound **1b** in DMSO-d_6

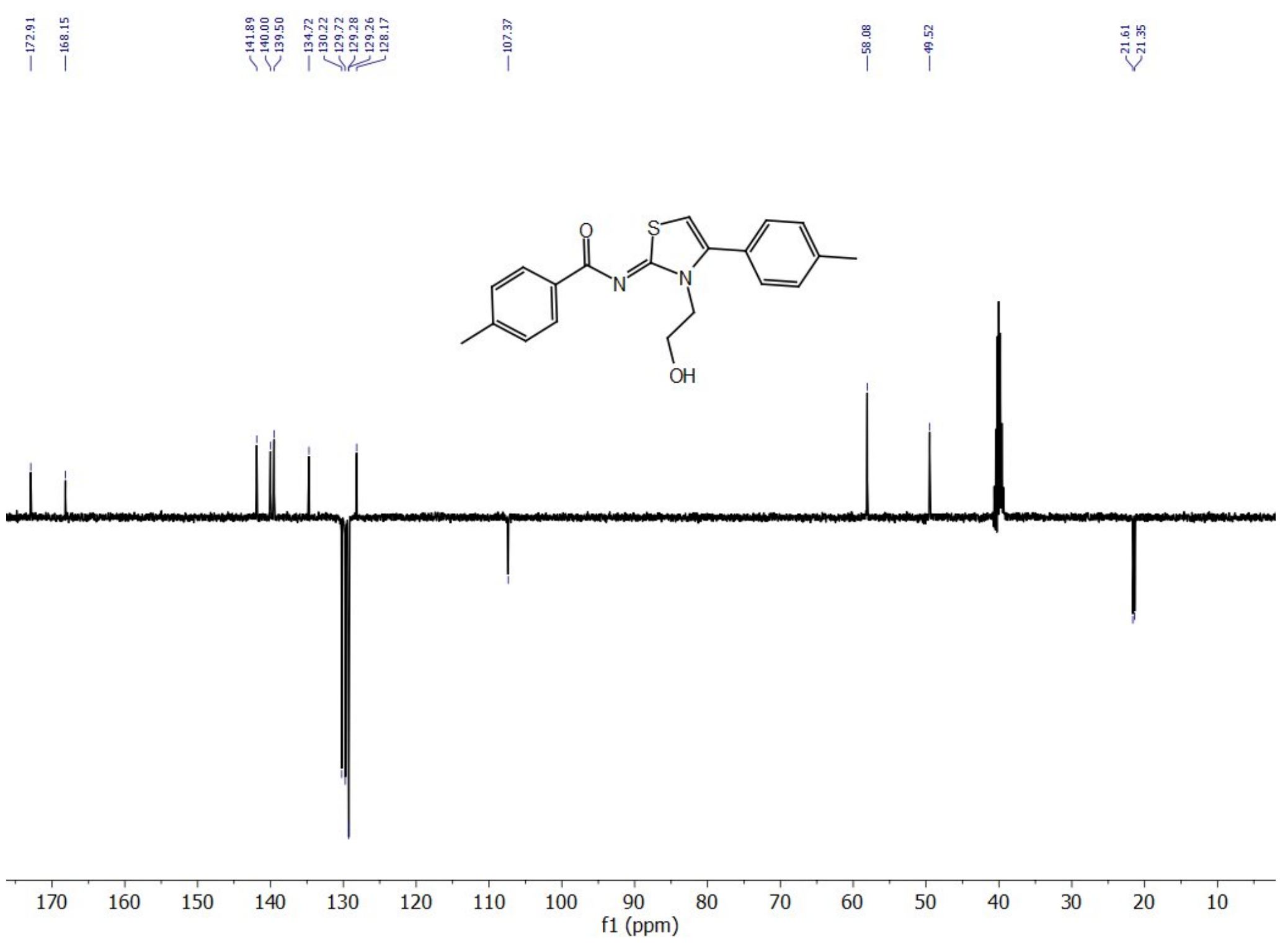

Figure S 6: ^{13}C-NMR APT spectrum for compound **1b** in DMSO-d_6

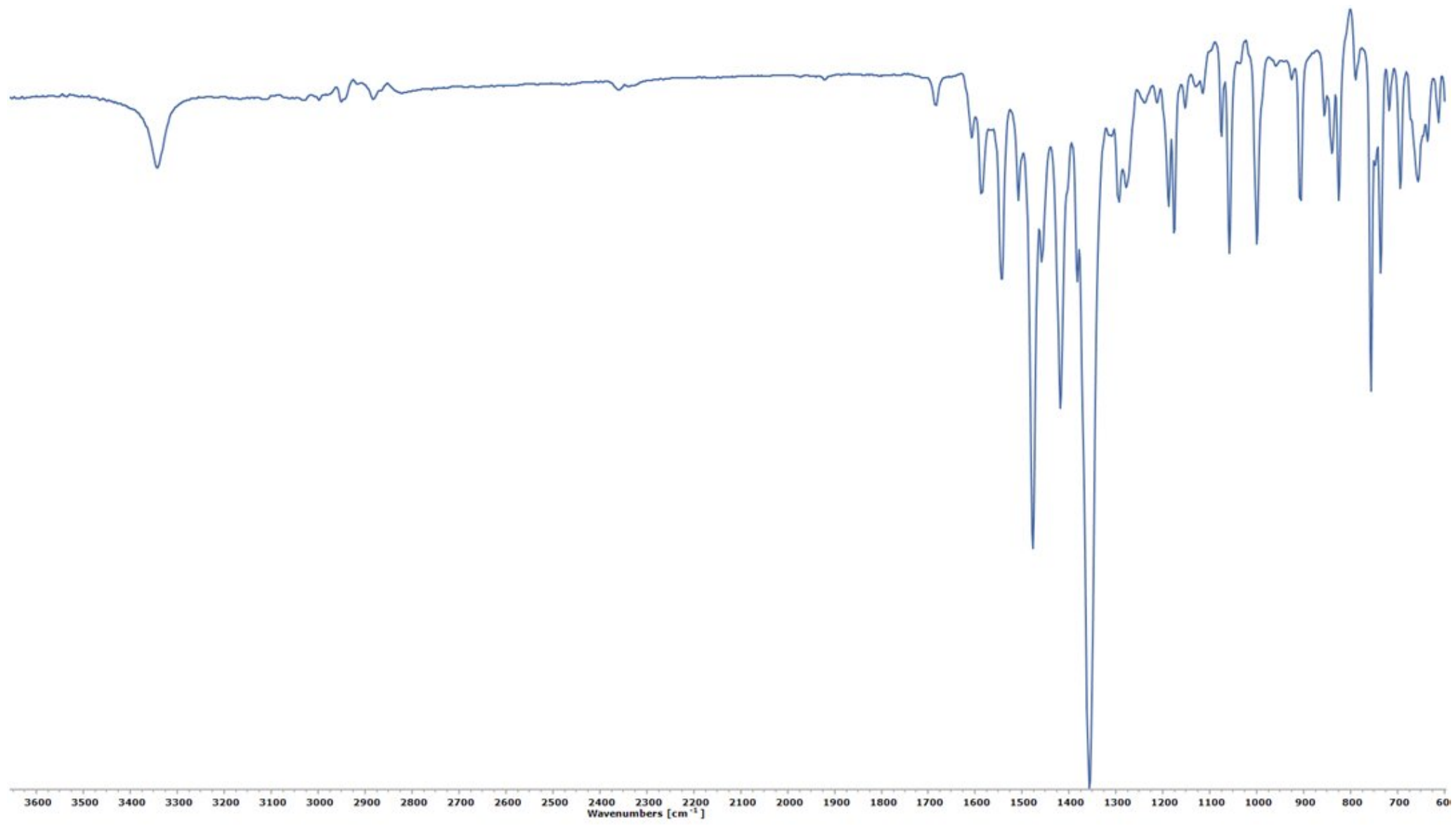

*Figure S 7: FTIR spectrum for compound **1b***

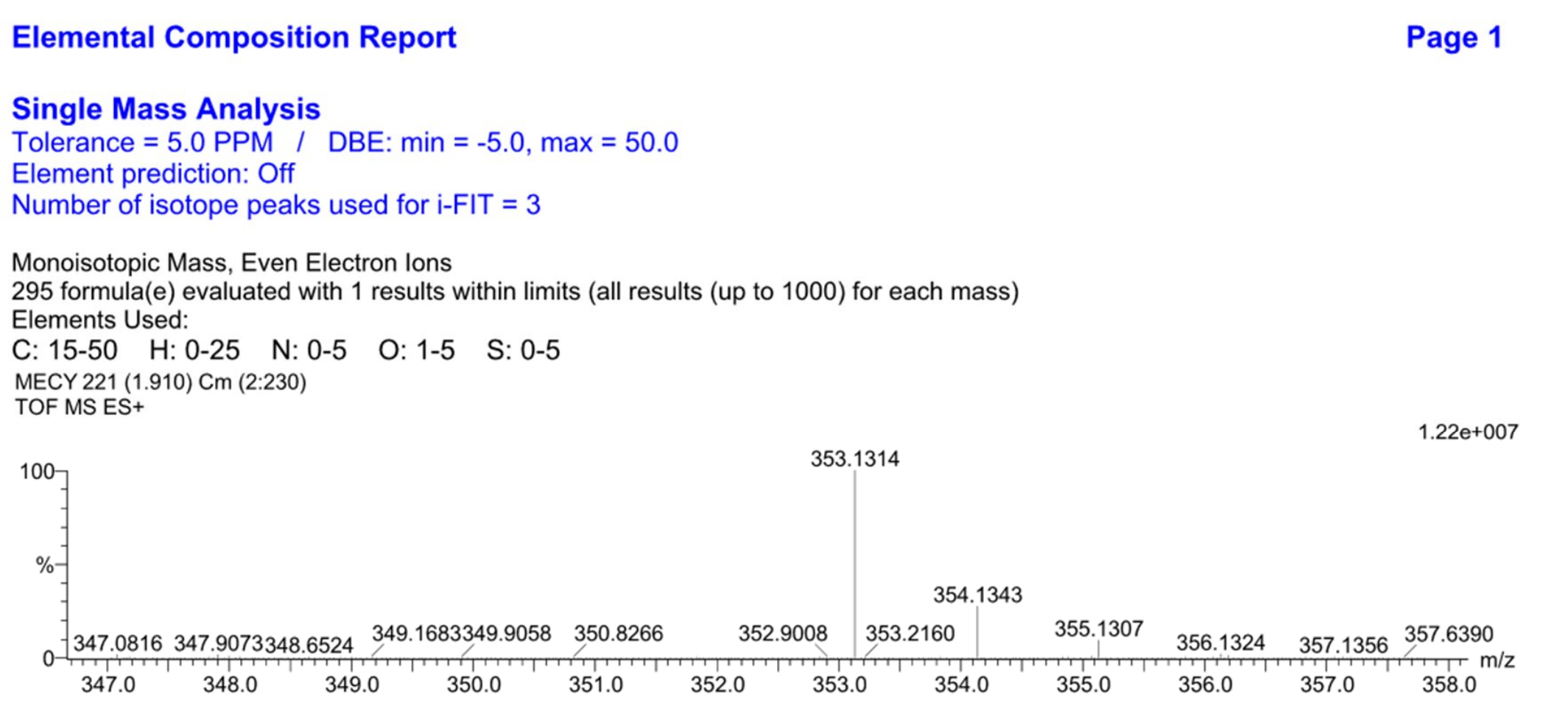

Minimum:				-5.0				
Maximum:		2.0	5.0	50.0				
Mass	Calc. Mass	mDa	PPM	DBE	i-FIT	Norm	Conf(%)	Formula
353.1314	353.1324	-1.0	-2.8	11.5	1095.4	n/a	n/a	C20 H21 N2 O2 S

*Figure S 8: HRMS spectrum for compound **1b** in methanol.*

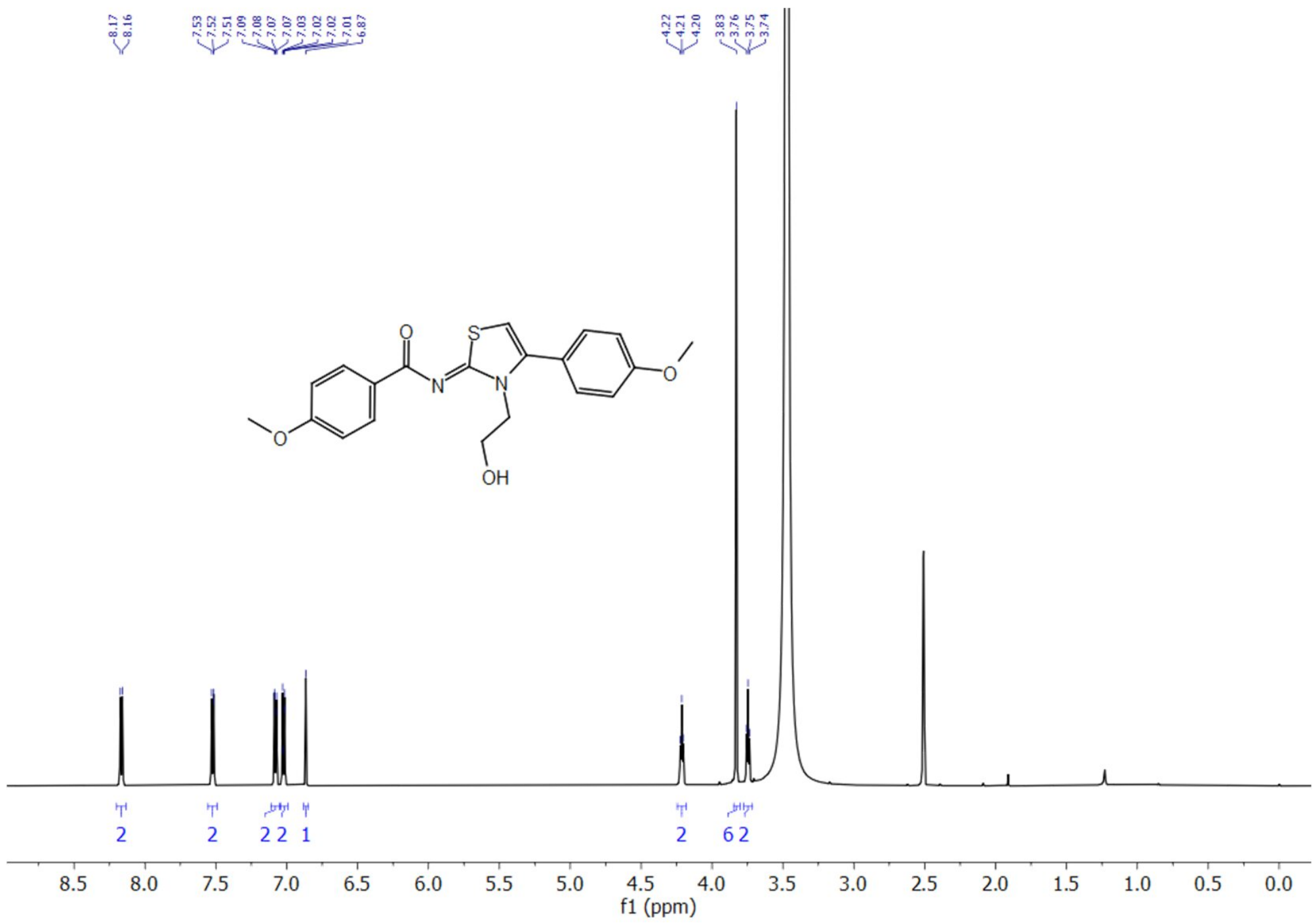

*Figure S 9: ^{1}H-NMR spectrum for compound **1c** in DMSO-d_6*

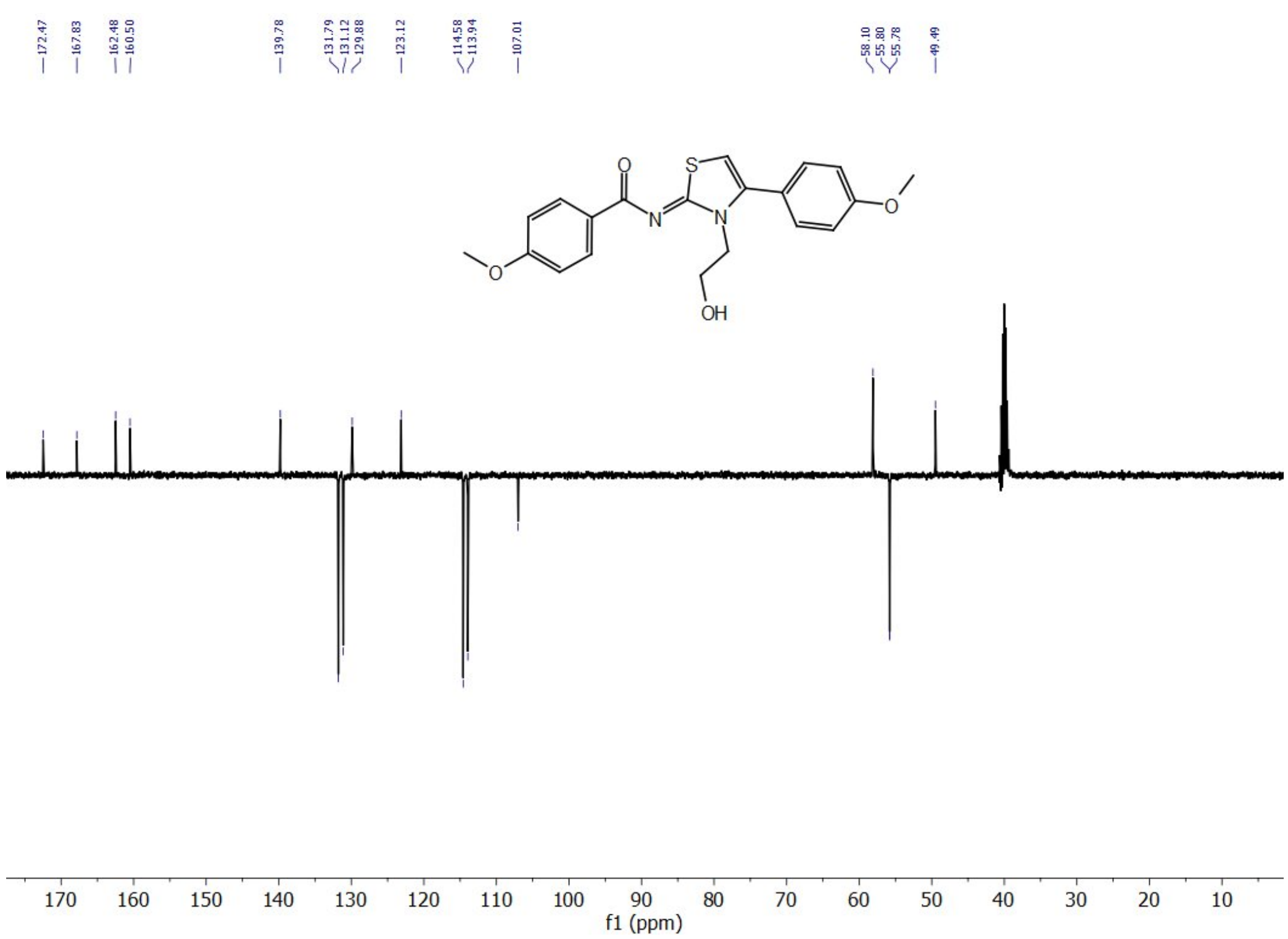

*Figure S 10: ^{13}C-NMR APT spectrum for compound **1c** in DMSO-d_6*

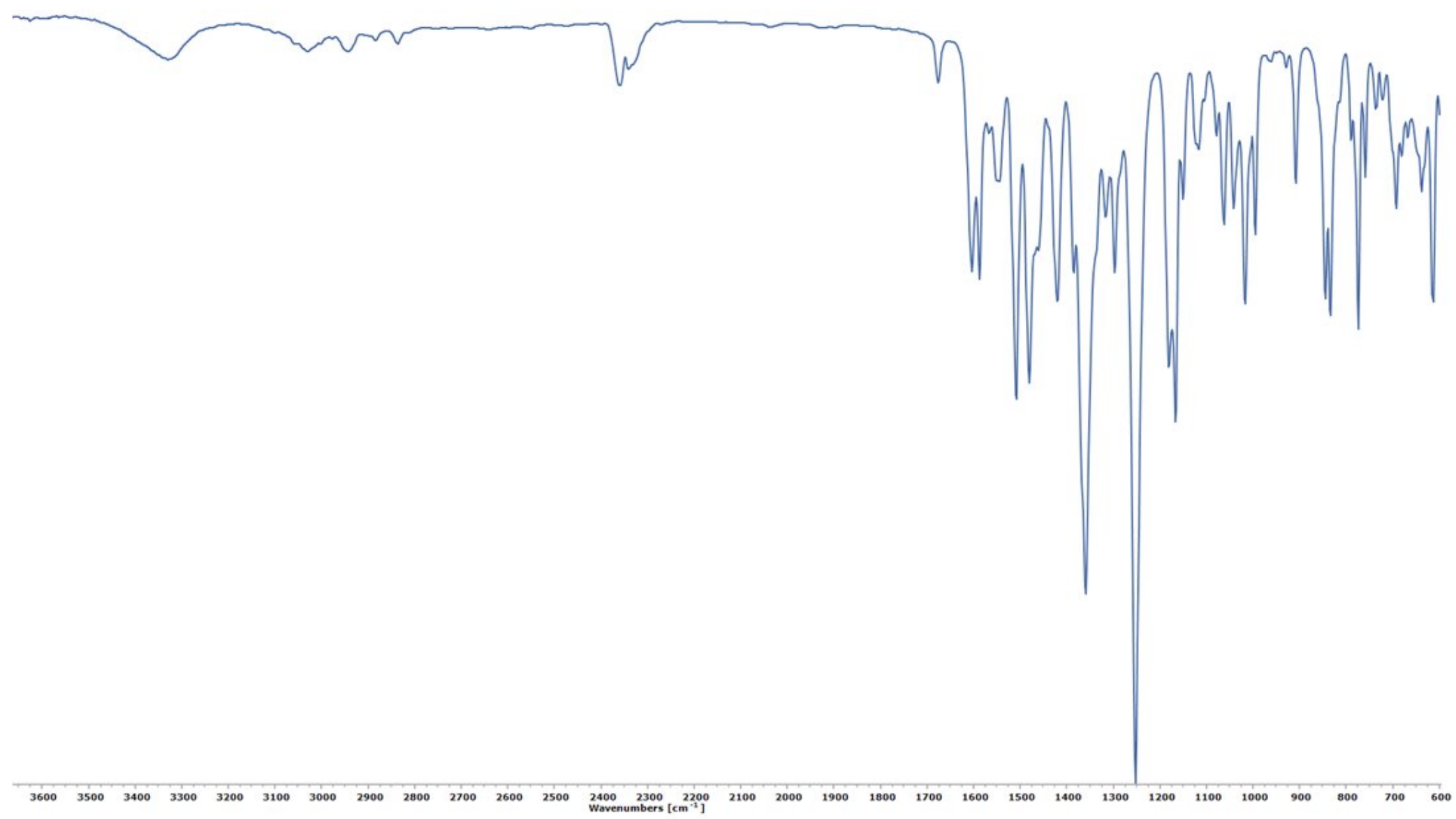

*Figure S 11: FTIR spectrum for compound **1c***

Elemental Composition Report **Page 1**

Single Mass Analysis
Tolerance = 5.0 PPM / DBE: min = -5.0, max = 50.0
Element prediction: Off
Number of isotope peaks used for i-FIT = 3

Monoisotopic Mass, Even Electron Ions
275 formula(e) evaluated with 1 results within limits (all results (up to 1000) for each mass)
Elements Used:
C: 15-50 H: 0-25 N: 1-5 O: 1-5 S: 0-5
MEOCY 141 (1.225) Cm (2:230)
TOF MS ES+

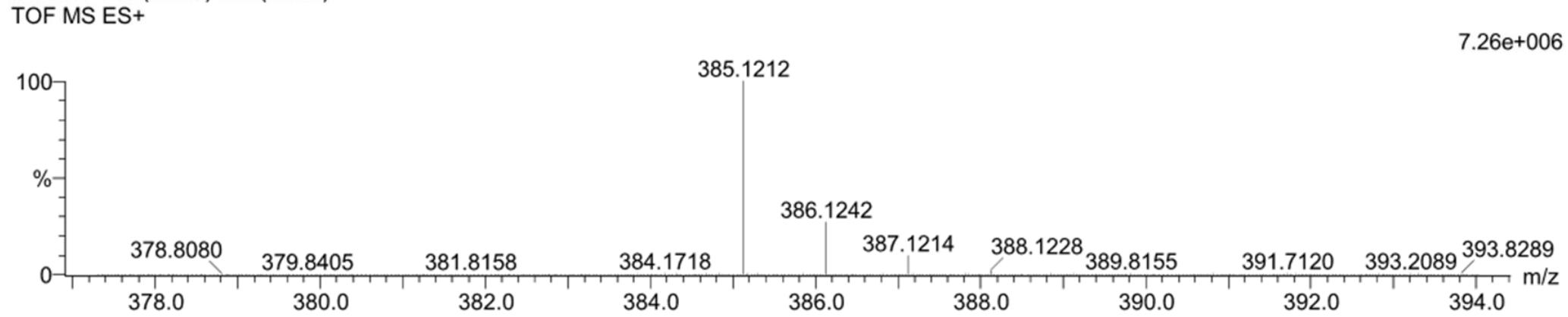

Mass	Calc. Mass	mDa	PPM	DBE	i-FIT	Norm	Conf(%)	Formula
Minimum:				-5.0				
Maximum:		2.0	5.0	50.0				
385.1212	385.1222	-1.0	-2.6	11.5	1100.4	n/a	n/a	C20 H21 N2 O4 S

*Figure S 12: HRMS spectrum for compound **1c** in methanol.*

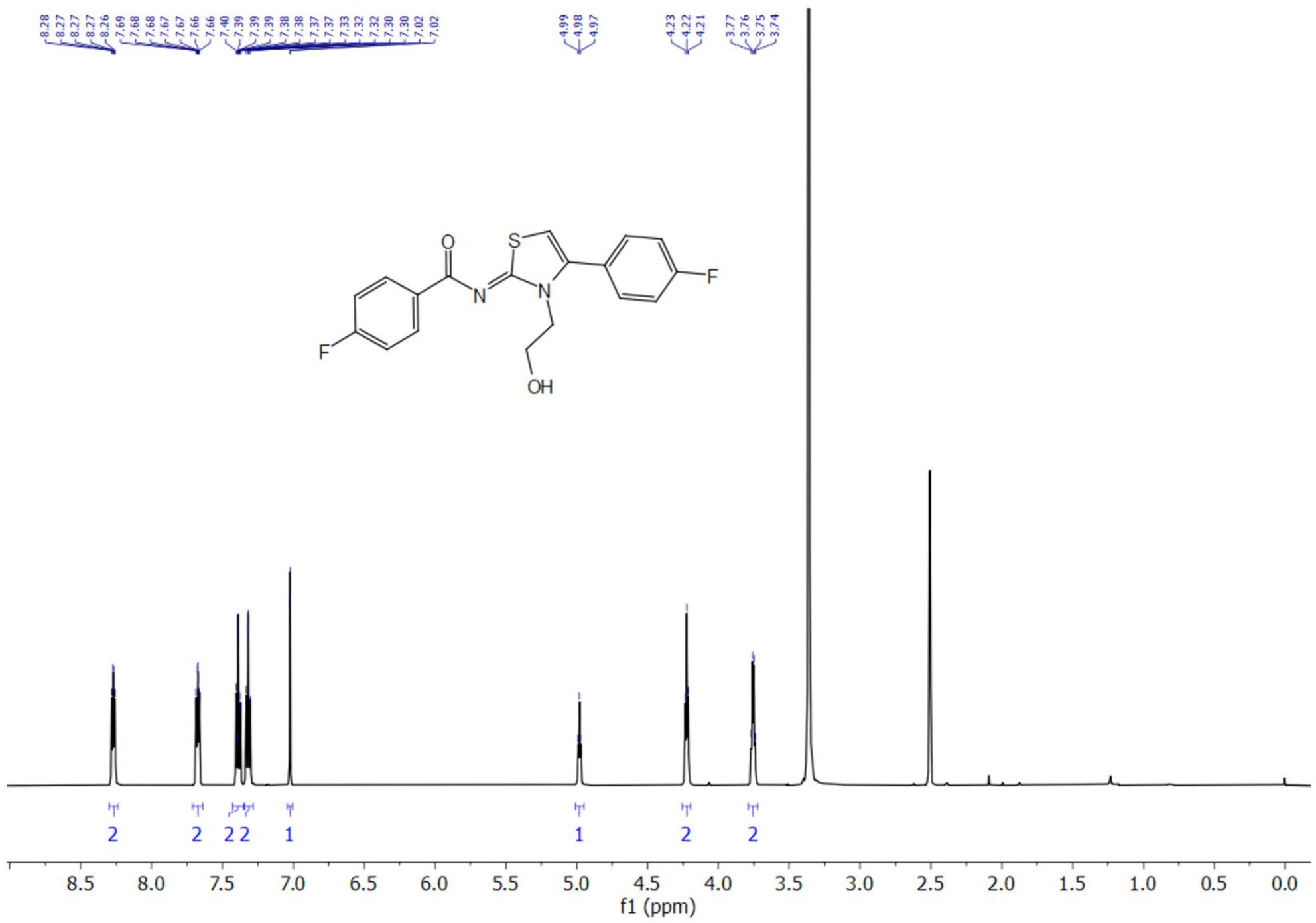

*Figure S 13: ¹H-NMR spectrum for compound **1d** in DMSO-d₆*

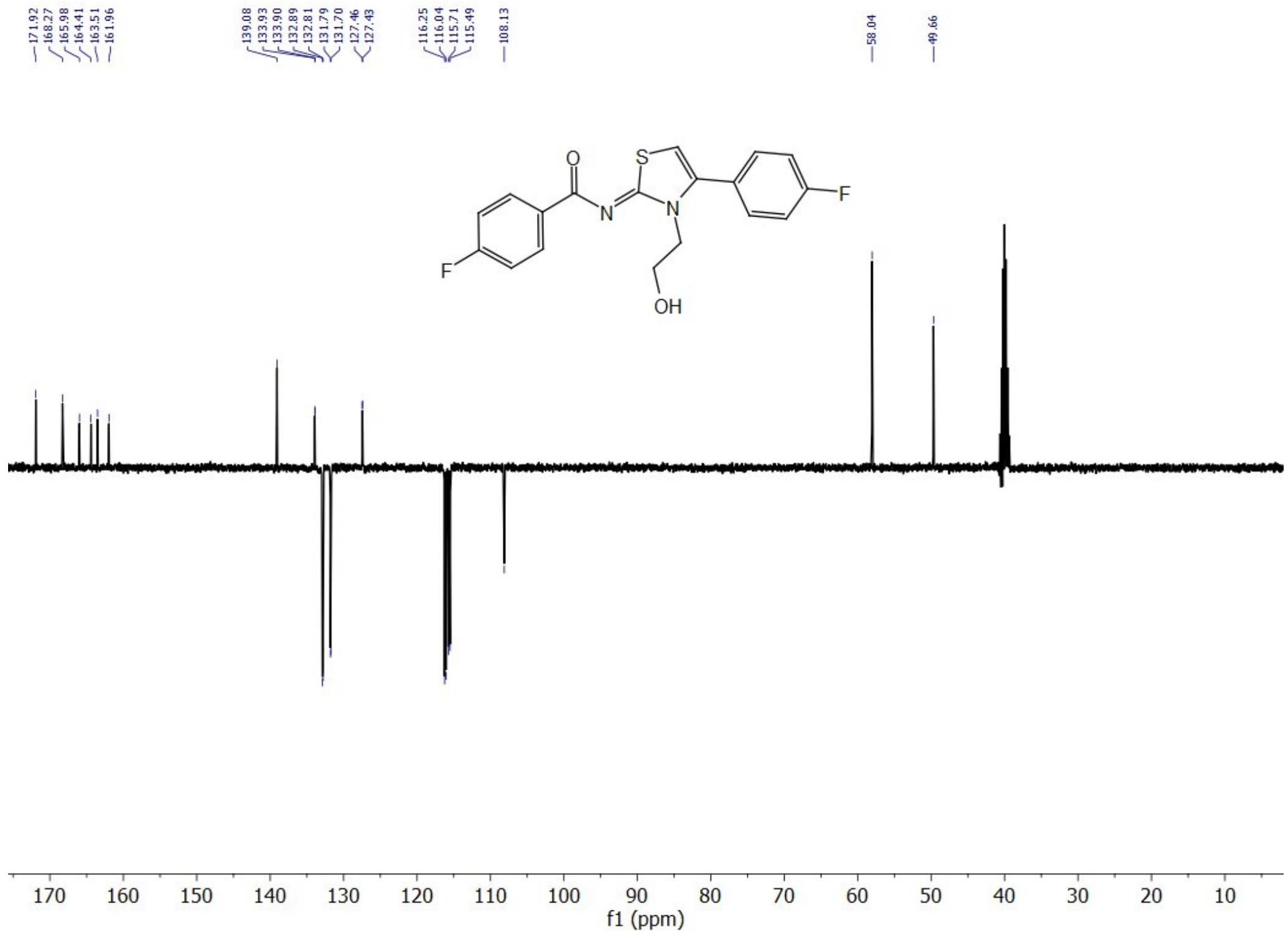

*Figure S 14: ¹³C-NMR APT spectrum for compound **1d** in DMSO-d₆*

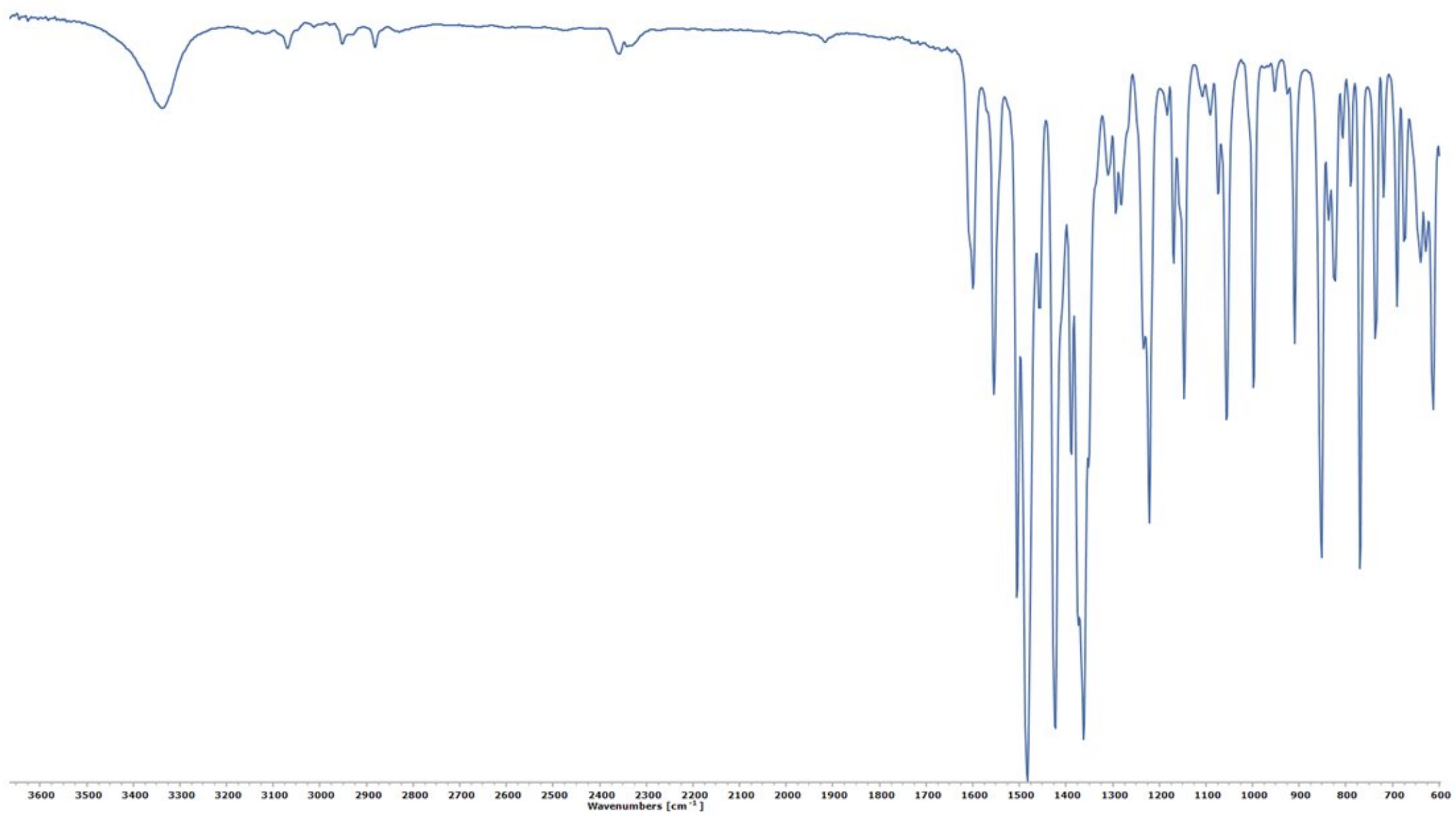

*Figure S 15: FTIR spectrum for compound **1d***

Elemental Composition Report **Page 1**

Single Mass Analysis
Tolerance = 5.0 PPM / DBE: min = -5.0, max = 50.0
Element prediction: Off
Number of isotope peaks used for i-FIT = 3

Monoisotopic Mass, Even Electron Ions
261 formula(e) evaluated with 1 results within limits (all results (up to 1000) for each mass)
Elements Used:
C: 15-50 H: 0-25 N: 1-5 O: 0-5 S: 0-5 F: 2-2

FICY 222 (1.919) Cm (2:230)
TOF MS ES+

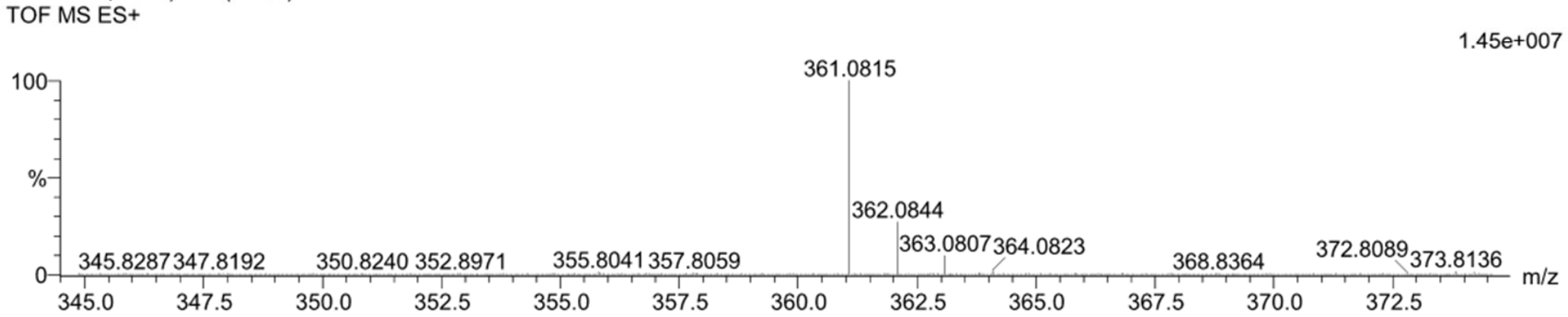

Mass	Calc. Mass	mDa	PPM	DBE	i-FIT	Norm	Conf(%)	Formula
Minimum:				-5.0				
Maximum:		2.0	5.0	50.0				
361.0815	361.0822	-0.7	-1.9	11.5	1201.7	n/a	n/a	C18 H15 N2 O2 S F2

*Figure S 16: HRMS spectrum for compound **1d** in methanol.*

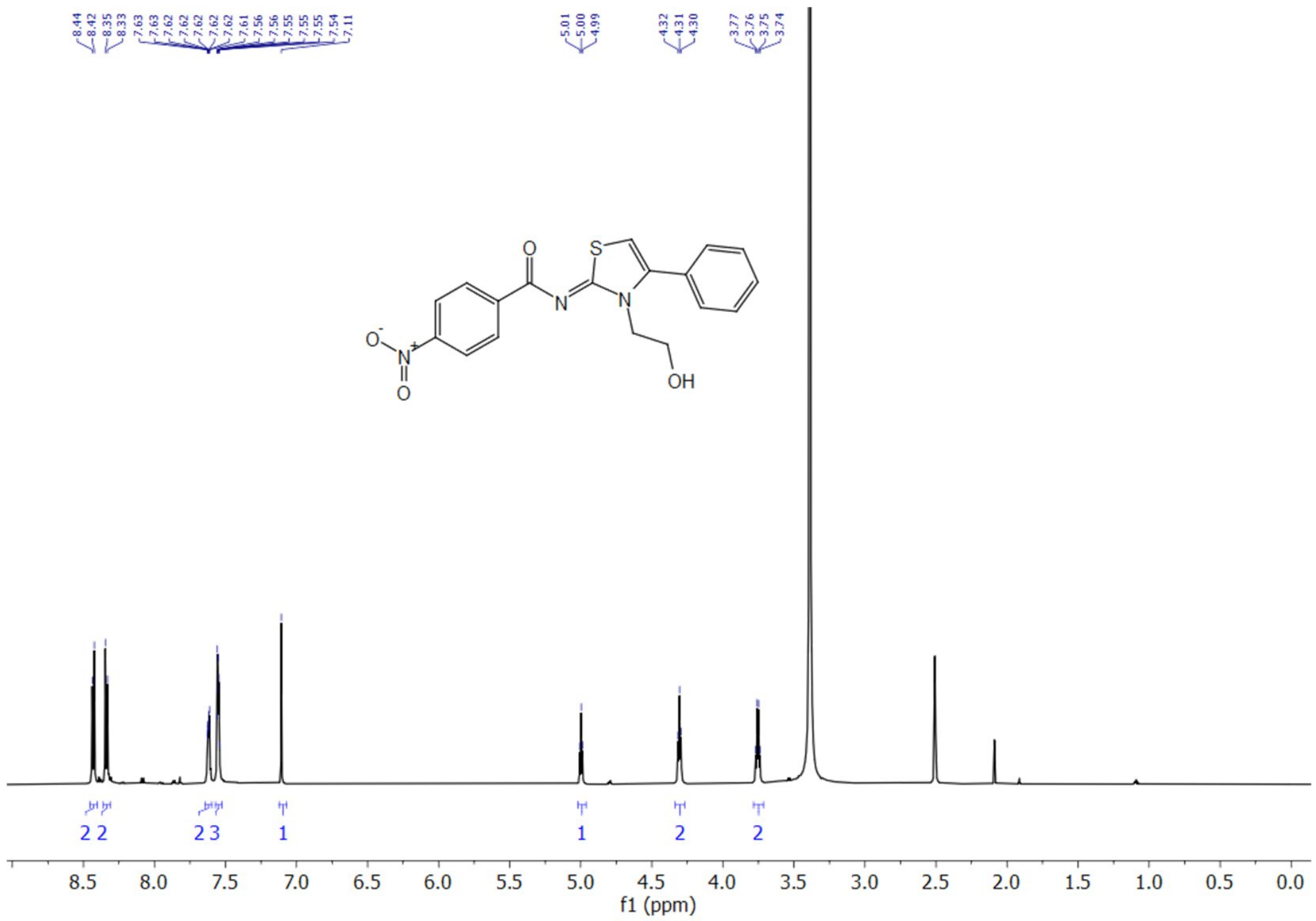

*Figure S 17: ^{1}H-NMR spectrum for compound **1e** in DMSO-d$_6$*

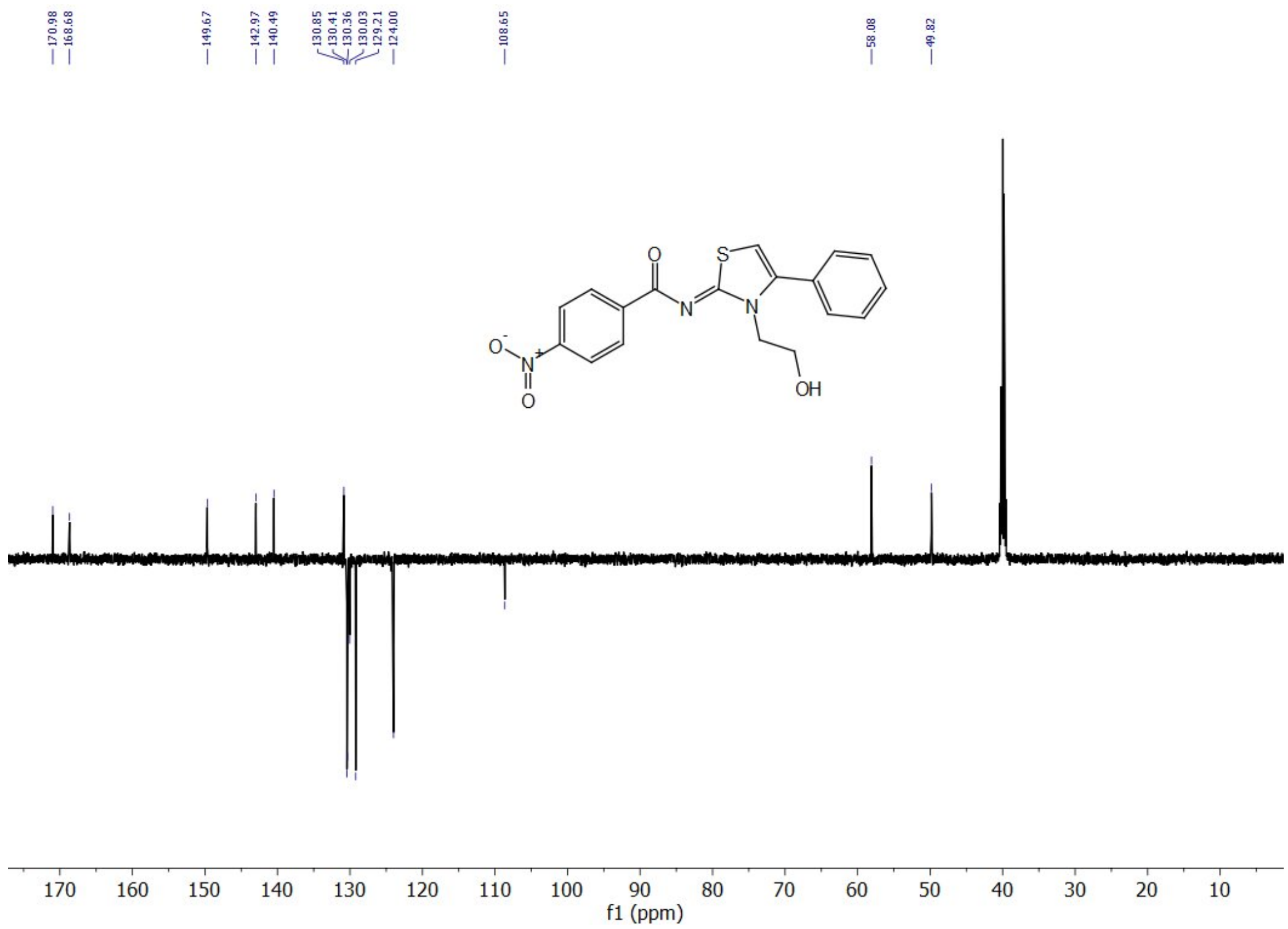

*Figure S 18: ^{13}C-NMR APT spectrum for compound **1e** in DMSO-d$_6$*

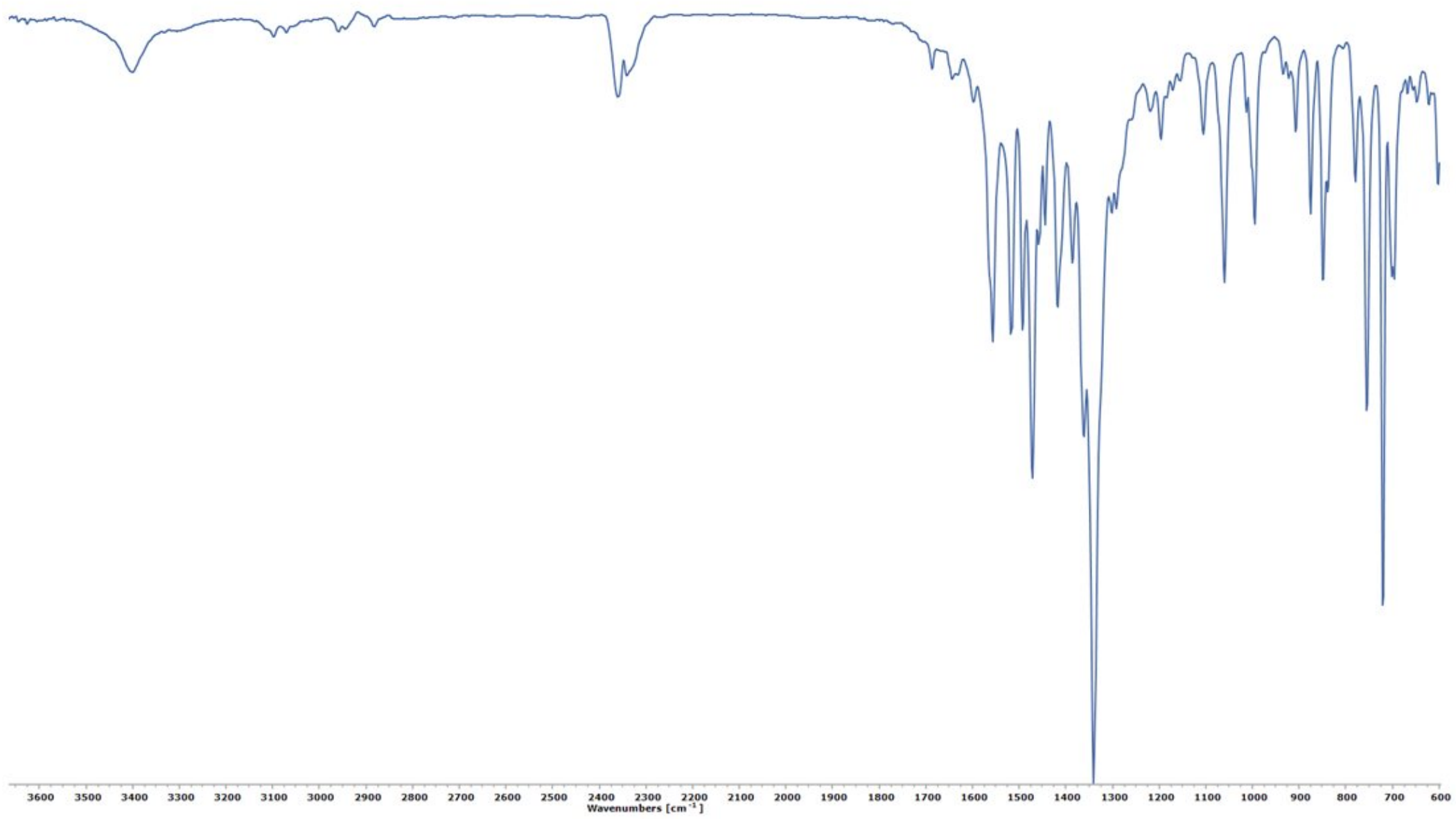

*Figure S 19: FTIR spectrum for compound **1e***

Elemental Composition Report **Page 1**

Single Mass Analysis
Tolerance = 5.0 PPM / DBE: min = -5.0, max = 50.0
Element prediction: Off
Number of isotope peaks used for i-FIT = 3

Monoisotopic Mass, Even Electron Ions
65 formula(e) evaluated with 1 results within limits (all results (up to 1000) for each mass)
Elements Used:
C: 15-50 H: 0-25 N: 1-5 O: 0-5 S: 1-1
NOUN 128 (1.113) Cm (2:230)
TOF MS ES+

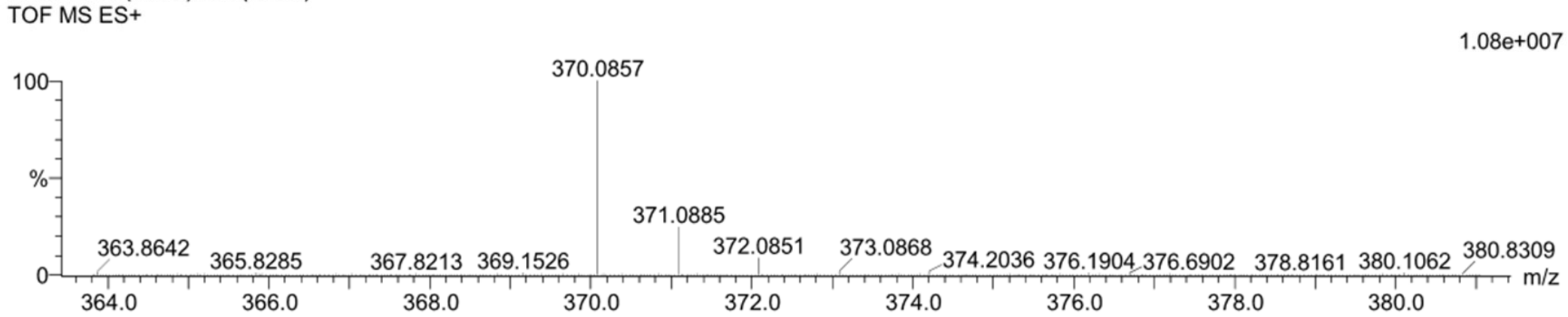

Minimum:				-5.0				
Maximum:		2.0	5.0	50.0				
Mass	Calc. Mass	mDa	PPM	DBE	i-FIT	Norm	Conf(%)	Formula
370.0857	370.0862	-0.5	-1.4	12.5	1094.6	n/a	n/a	C18 H16 N3 O4 S

*Figure S 20: HRMS spectrum for compound **1e** in methanol.*

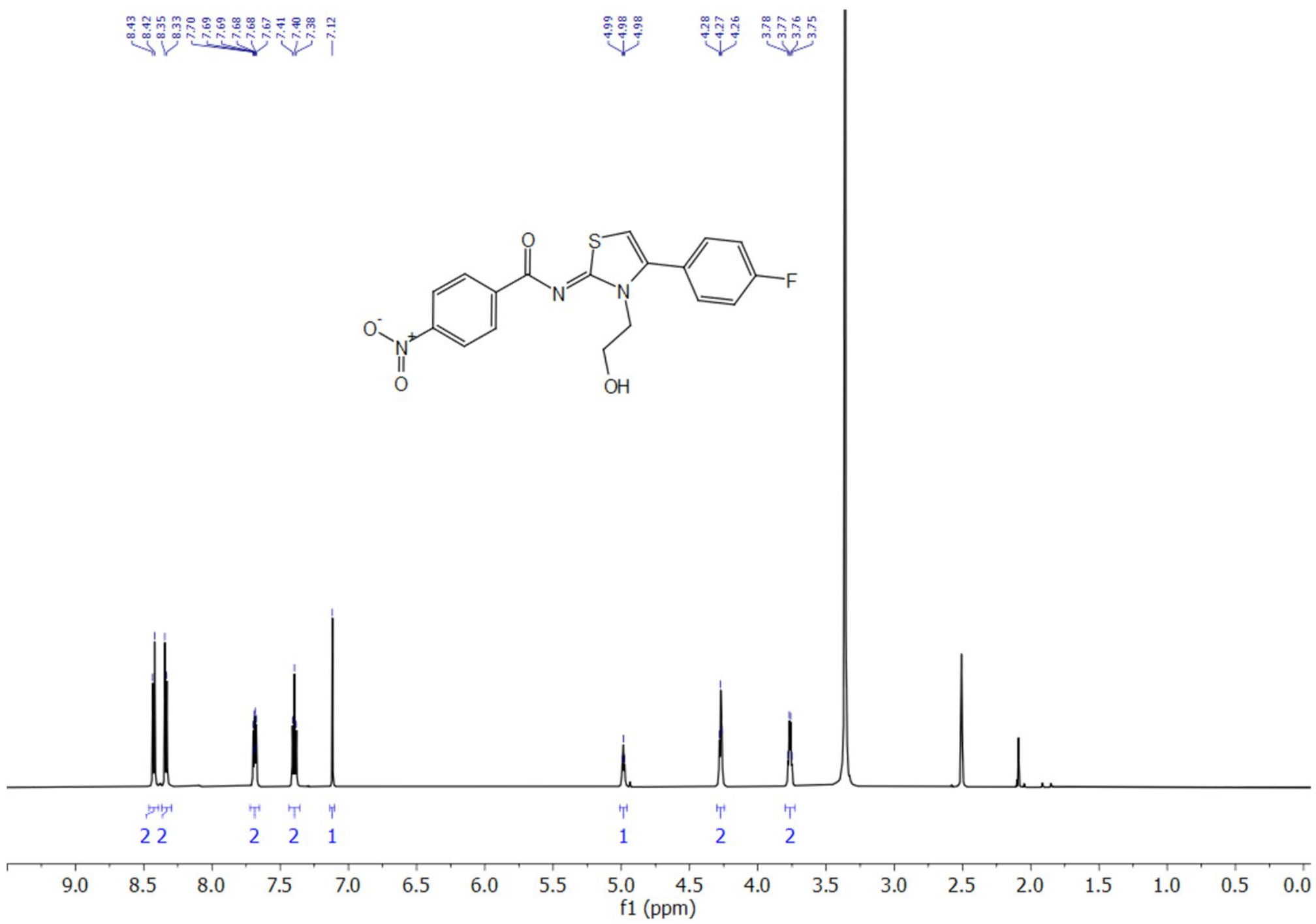

*Figure S 21: ^{1}H-NMR spectrum for compound **1f** in DMSO-d$_6$*

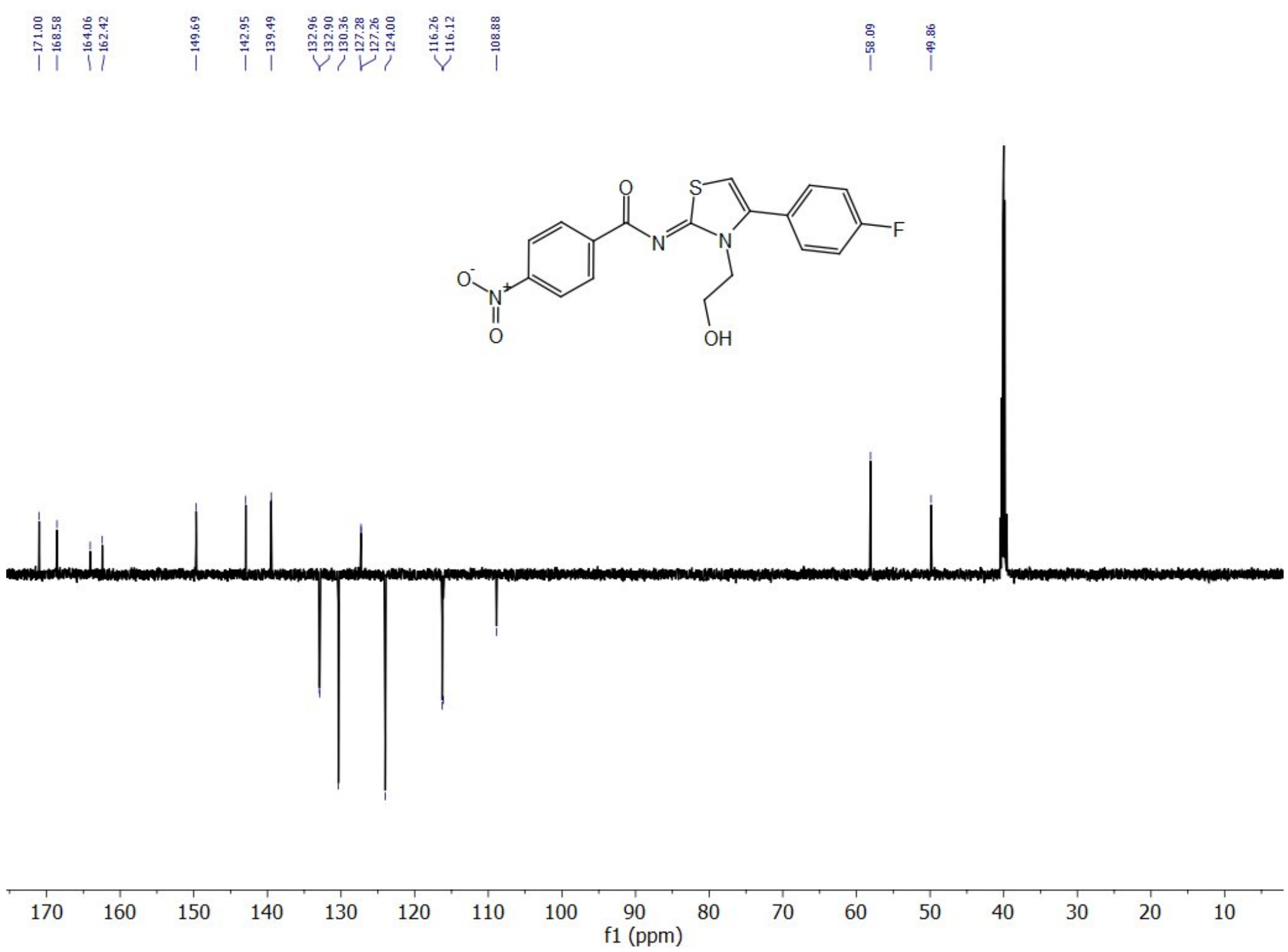

*Figure S 22: ^{13}C-NMR APT spectrum for compound **1f** in DMSO-d$_6$*

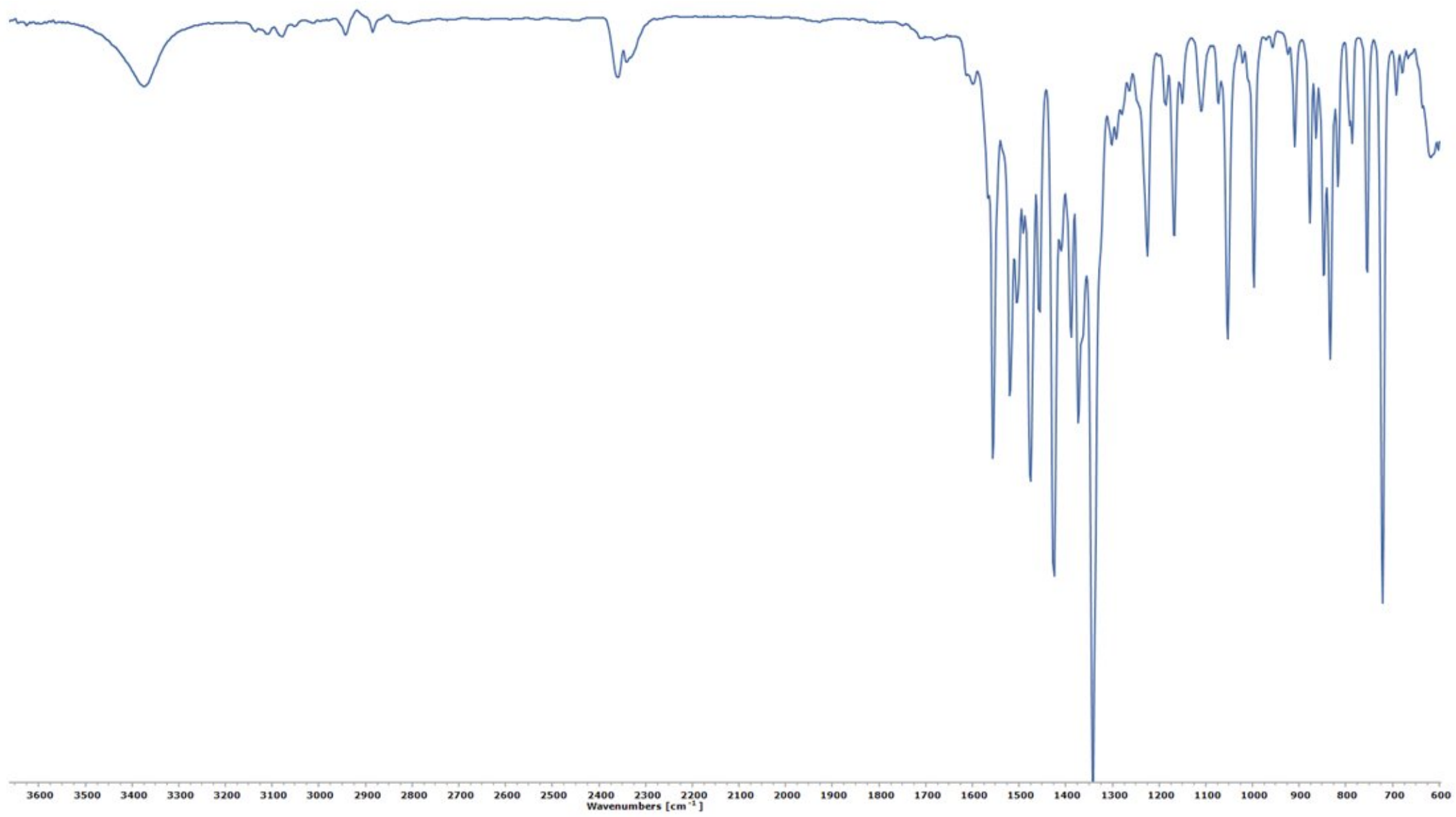

*Figure S 23: FTIR spectrum for compound **1f***

Elemental Composition Report **Page 1**

Single Mass Analysis
Tolerance = 5.0 PPM / DBE: min = -5.0, max = 50.0
Element prediction: Off
Number of isotope peaks used for i-FIT = 3

Monoisotopic Mass, Even Electron Ions
69 formula(e) evaluated with 1 results within limits (all results (up to 1000) for each mass)
Elements Used:
C: 15-50 H: 0-25 N: 1-5 O: 0-5 S: 1-1 F: 1-1

NOFI 121 (1.053) Cm (2:230)
TOF MS ES+

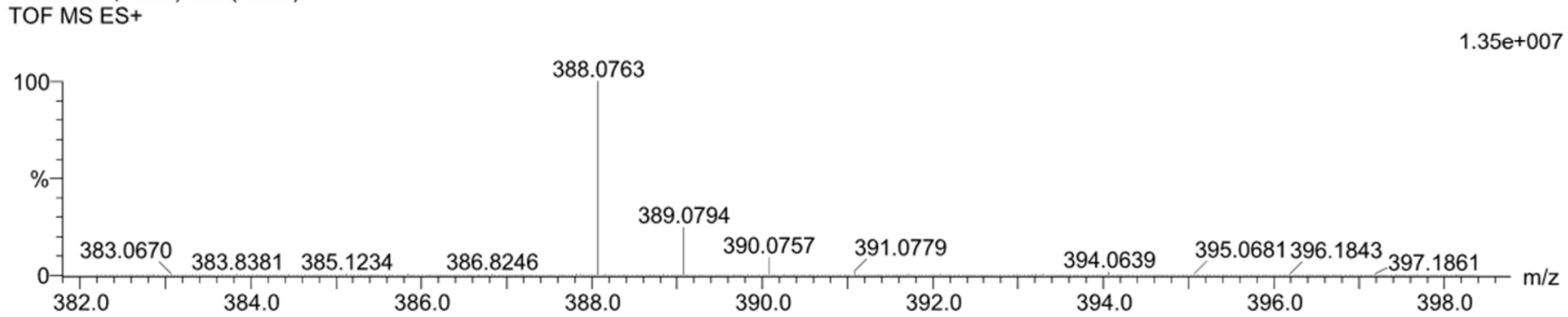

	Calc. Mass	mDa	PPM	DBE	i-FIT	Norm	Conf(%)	Formula
Minimum:				-5.0				
Maximum:		2.0	5.0	50.0				
Mass	Calc. Mass	mDa	PPM	DBE	i-FIT	Norm	Conf(%)	Formula
388.0763	388.0767	-0.4	-1.0	12.5	1138.0	n/a	n/a	C18 H15 N3 O4 S F

*Figure S 24: HRMS spectrum for compound **1f** in methanol.*

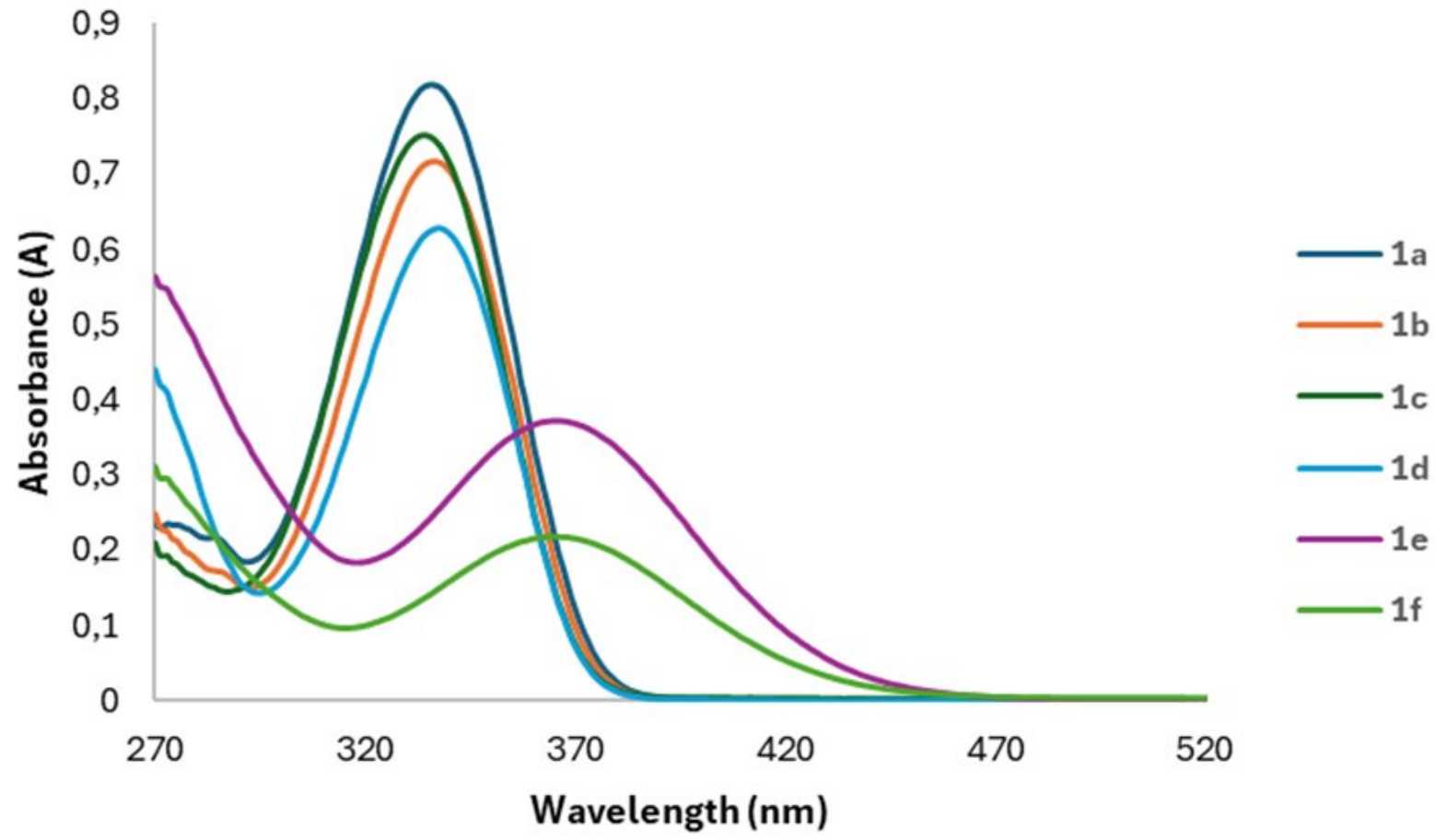

*Figure S 25: UV-Vis spectra for compounds **1a-1f** measured in DMSO.*

Single-crystal XRD - Crystallography.

*Table S 1: Crystal data collection and refinement parameters for compounds **1b, 1c** and **1f**.*

	1b	1c	1f
Empirical formula	$C_{20}H_{20}N_2O_2S$	$C_{18}H_{14}F_2N_2O_2S$	$C_{18}H_{14}N_3O_4FS$
Formula weight	352.44	360.37	387.38
Crystal size (mm^{-3})	0.22 x 0.25 x 0.26	0.227 x 0.193 x 0.15	0.247 x 0.192 x 0.09
Temp (K)	173	173	173
Radiation (Å)	CuKα (λ = 1.54178)	CuKα (λ = 1.54178)	CuKα (λ = 1.54178)
Crystal system	monoclinic	Triclinic	Triclinic
Space group	P*21/n*	P-*1*	P-*1*
a (Å)	11.5222(6)	8.4805(3)	9.0972(3)
b (Å)	12.0640(6)	9.4282(3)	9.1986(3)
c (Å)	12.8391(6)	11.1622(4)	11.2375(4)
α (°)	90	98.7460(10)	98.4410(10)
β (°)	99.729(3)	111.9680(10)	113.5900(10)
γ (°)	90	99.6030(10)	96.428(2)
Volume ($Å^3$)	1759.02(15)	793.32(5)	837.20(5)
Z	4	2	2
Density (calc) (kg/m^3)	1.331	1.509	1.537
Abs. coeff. (mm^{-1})	3.447	2.155	2.109
Index ranges	$-14 \le h \le 11$	$-10 \le h \le 10$	$-11 \le h \le 11$
	$-14 \le k \le 14$	$-11 \le k \le 11$	$-11 \le k \le 11$
	$-15 \le l \le 15$	$-13 \le l \le 13$	$-13 \le l \le 13$
F(000)	744	372.0	400.0
Parameters	229	227	248
Reflections collected	29685	28606	25074
Independent reflections	3349[R_{int} = 0.044]	2985 [Rint = 0.0405]	3168 [R_{int} = 0.0428]
Goodness-of-fit on F^2	1.053	1.101	1.076
R_1 [$I > 2\sigma(I)$]	0.0387	0.0388	0.0384
R_1 (all data)	0.0427	0.0404	0.0403
wR_2 [$I > 2\sigma(I)$]	0.1022	0.0990	0.0960
wR_2 [all data]	0.1022	0.1004	0.0976
Large diff. peak hole (e.$Å^{-3}$)	0.163 and -0.346	0.28 and -0.49	0.22 and -0.47

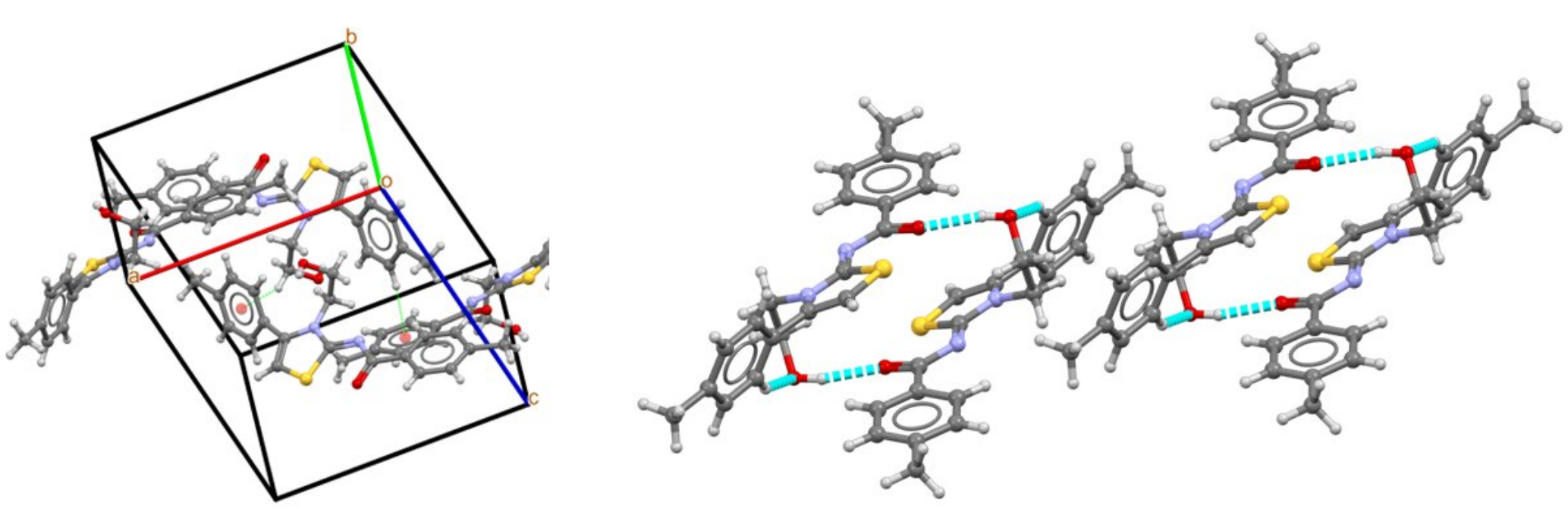

(a) (b)

*Figure S 26: Crystal packing for compound **1b** showing (a) intermolecular π-π interactions and (b) intermolecular hydrogen bonding networks*

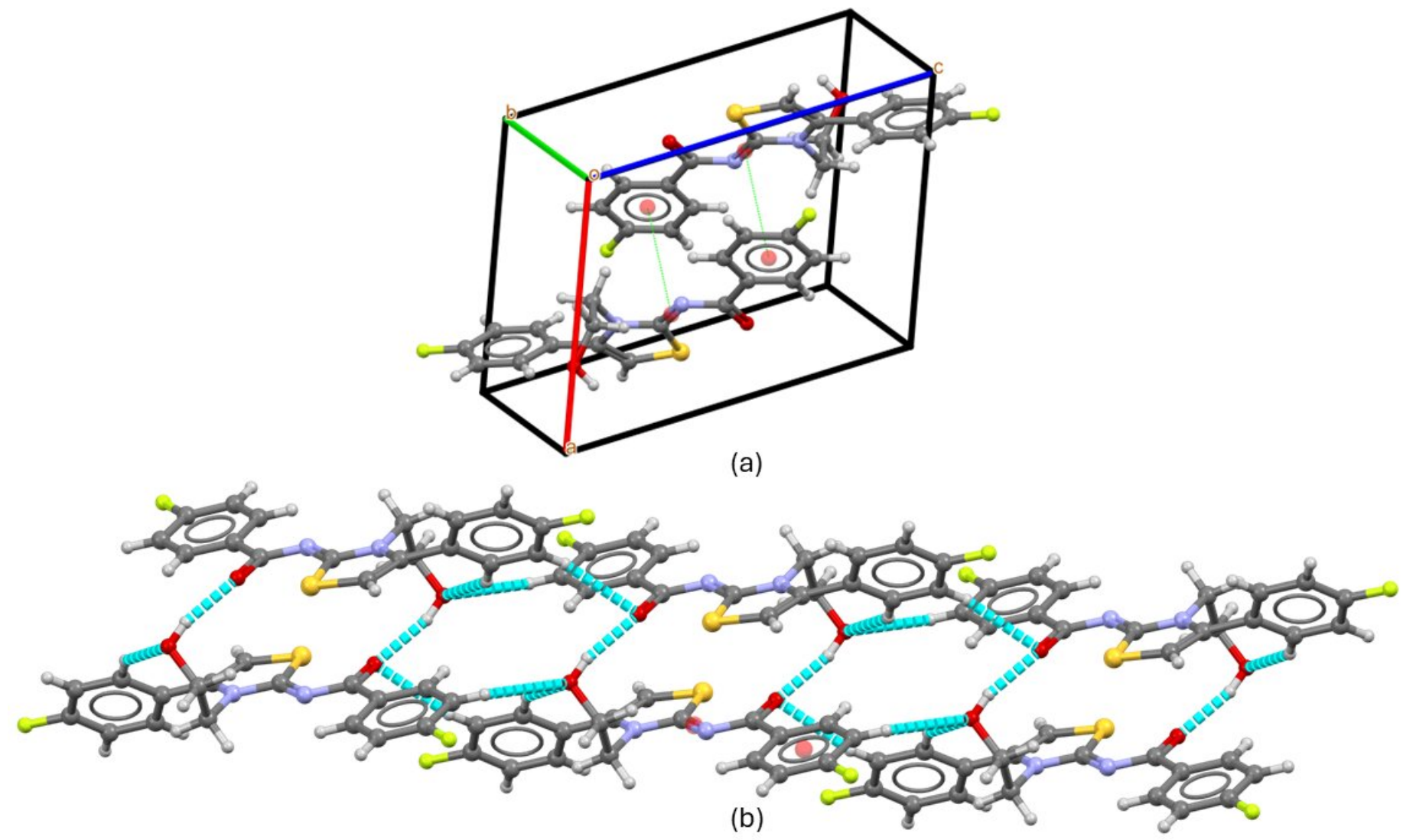

*Figure S 27: Crystal packing for compound **1c** showing (a) intermolecular π-π interactions and (b) intermolecular hydrogen bonding networks*

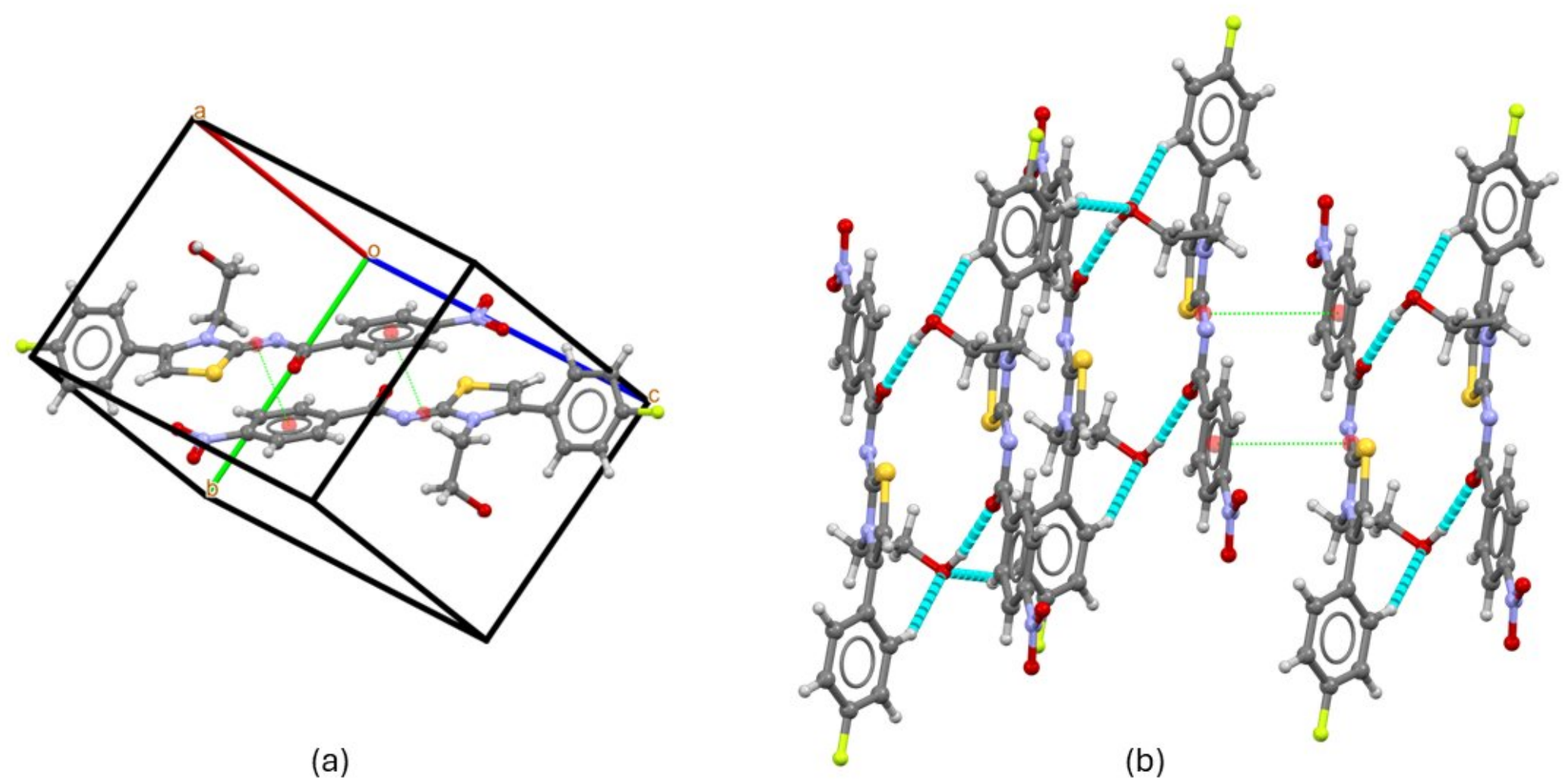

*Figure S 28: Crystal packing for compound **1f** showing (a) intermolecular π-π interactions and (b) intermolecular hydrogen bonding networks*

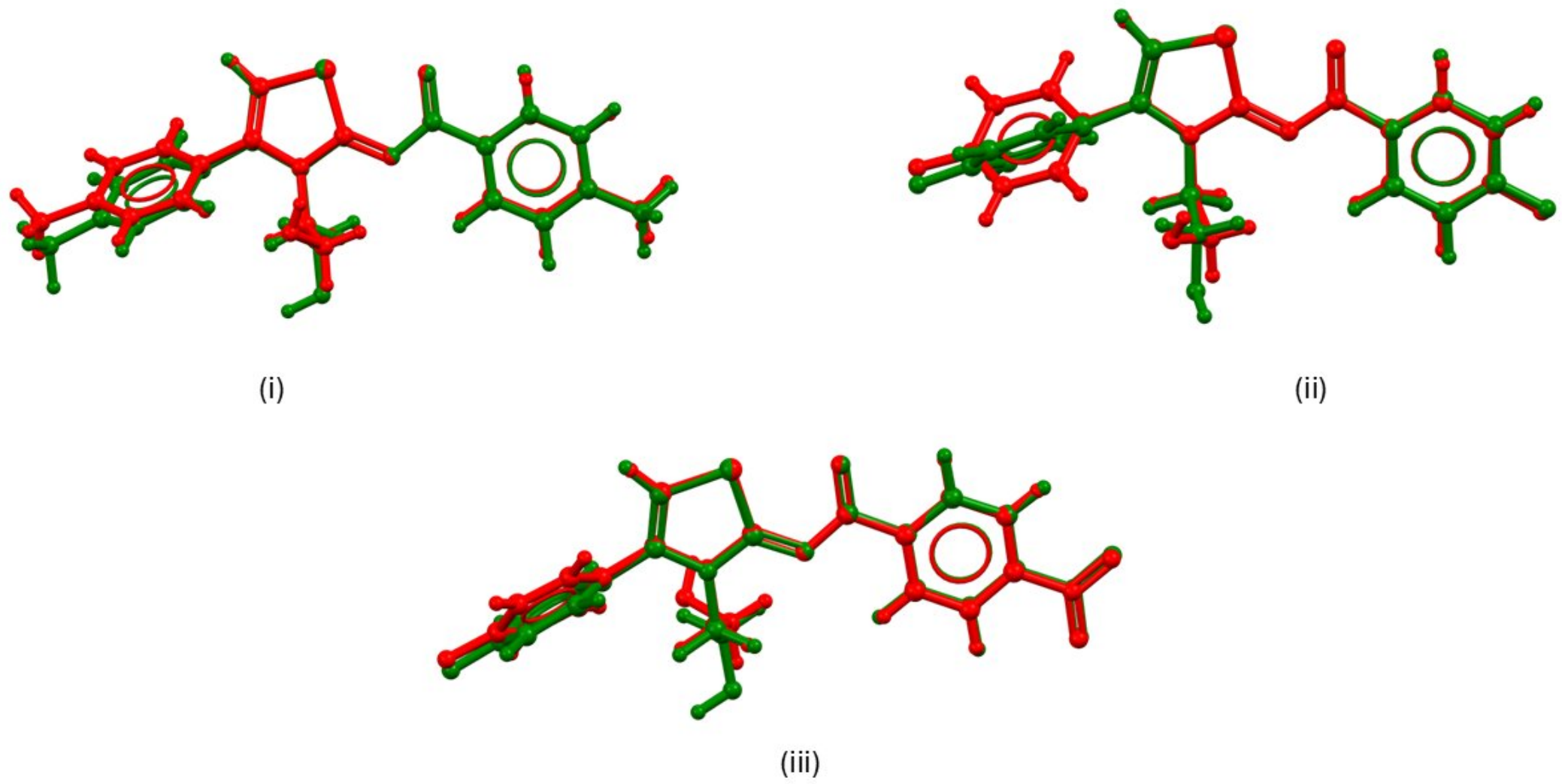

*Figure S 29: Overlay of structures (crystal structures and calculated structures)) for compounds **1b, 1c**, and **1f***

*Table S 2: Selected bond lengths for compounds **1a-1f***

	Bond lengths (Å)					
	**1b** (calculated)	**1b** (experimental)	**1c** (calculated)	**1c** (experimental)	**1f** (calculated)	**1f** (experimental)
**C-O**	1.23450	1.2510(19)	1.23379	1.250(2)	1.23289	1.2480(19)
**(O)C-N**	1.38011	1.359(2)	1.37825	1.3551(19)	1.37326	1.3497(19)
**N-C(S)$_{(imine)}$**	1.30351	1.327(2)	1.30495	1.319(2)	1.30818	1.325(2)
**(S)C-N$_{(thiazole)}$**	1.37214	1.359(2)	1.37116	1.3606(19)	1.36832	1.3571(19)
**(C)N-C$_{(thiazole)}$**	1.40567	1.404(2)	1.40547	1.4018(19)	1.40456	1.404(2)
**(N)C-C$_{(thiazole)}$**	1.34875	1.340(2)	1.34886	1.343(2)	1.34814	1.351(2)
**(C)C-S$_{(thiazole)}$**	1.75477	1.7247(17)	1.75346	1.7243(16)	1.75327	1.7232(16)
**(N)C-S$_{(thiazole)}$**	1.77258	1.7343(16)	1.77126	1,7378(15)	1.76849	1.7341(16)

Computational details.

Molecular docking was done using AutoDock Vina *1.2.0*. Protein–ligand interactions were inspected and visualised using Biovia Discovery Studio Visualizer *v*21. All DFT calculations were performed using *Gaussian 16*. Visualization of the molecular orbitals and molecular electrostatic potential was done using the GaussView 6 program. The XYZ coordinates for the optmised (in gas) compounds 1a-1f are presented below.

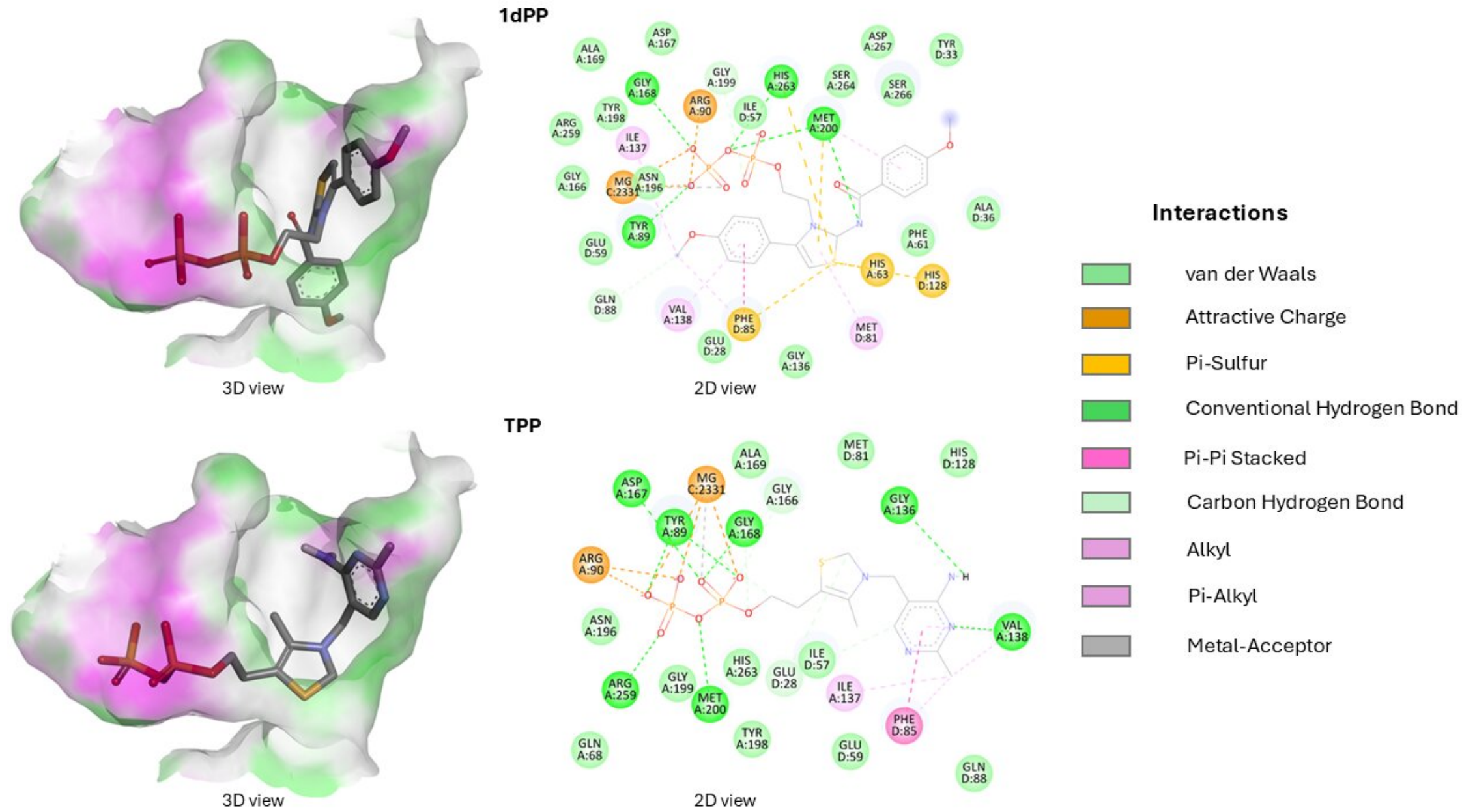

*Figure S 30: A comparison of the best docking poses for **1aPP** and native **TPP** on human pyruvate dehydrogenase*

*Table S3: XYZ coordinates for the optimized gas-phase geometry of **1a***

1a optimized geometry (B3LYP/6-311++G(d,p))

S	0.0877500000	-2.4704660000	-0.2391340000
O	0.8511060000	3.4636400000	-0.8869460000
O	-2.5035260000	-2.1214010000	-0.1933420000
N	0.9595030000	-0.0682140000	0.1500410000
N	-1.3754460000	-0.1376220000	0.1181160000
C	3.9133080000	0.2570750000	1.3609480000
C	5.2167800000	0.7270180000	1.4904350000
C	6.1059610000	0.6328150000	0.4198800000
C	5.6852600000	0.0641130000	-0.7800040000
C	4.3786320000	-0.4023600000	-0.9136750000
C	3.4774940000	-0.3088960000	0.1540050000
C	0.8057770000	2.0574130000	-1.1198530000
C	0.9574510000	1.3967100000	0.2486760000
C	1.8134690000	-2.1616810000	-0.1986690000
C	2.1103770000	-0.8634610000	0.0170240000
C	-3.8449440000	1.2290950000	0.3328570000
C	-5.0671650000	1.8896390000	0.4282840000
C	-6.2594470000	1.1783660000	0.2951590000
C	-6.2263950000	-0.1981250000	0.0666760000
C	-5.0065890000	-0.8596430000	-0.0281270000
C	-0.2256350000	-0.7491380000	0.0384390000

C	-2.5159630000	-0.9021260000	-0.0045290000
C	-3.8054880000	-0.1515610000	0.1037000000
H	3.2351590000	0.3096870000	2.2054710000
H	5.5415010000	1.1576760000	2.4307730000
H	7.1213140000	0.9981310000	0.5229000000
H	6.3716560000	-0.0139280000	-1.6154740000
H	4.0488500000	-0.8379540000	-1.8499030000
H	0.6409460000	3.9241680000	-1.7042960000
H	1.6249070000	1.7410360000	-1.7787270000
H	-0.1471420000	1.7563960000	-1.5668510000
H	0.1259350000	1.6872610000	0.8884350000
H	1.8892860000	1.7200910000	0.7070770000
H	2.5247110000	-2.9671250000	-0.2886020000
H	-2.9142870000	1.7712200000	0.4363990000
H	-5.0902570000	2.9589300000	0.6074520000
H	-7.2107480000	1.6938830000	0.3699220000
H	-7.1520960000	-0.7533250000	-0.0365640000
H	-4.9600750000	-1.9272620000	-0.2040110000

Table S4: XYZ coordinates for the optimized gas-phase geometry of ***1b***

1b optimized geometry (B3LYP/6-311++G(d,p))

S	-0.1173120000	2.6201160000	-0.2157720000
O	-0.8033600000	-3.3262300000	-1.1105250000
O	2.4758000000	2.3067710000	-0.1549100000
N	-0.9580600000	0.1986010000	0.1233780000
N	1.3755250000	0.2989460000	0.0951770000
C	-7.5118110000	-1.1663270000	0.4544550000
C	7.6328280000	-1.6624510000	0.3343200000
C	-4.3674500000	0.4723550000	-0.9659020000
C	-5.6659500000	-0.0215350000	-0.8506000000
C	-6.1153580000	-0.6073780000	0.3353570000
C	-5.2219370000	-0.6733620000	1.4129090000
C	-3.9273370000	-0.1787390000	1.3069400000
C	-3.4771550000	0.3981930000	0.1100790000
C	-0.8111950000	-1.9056790000	-1.2000260000
C	-0.9387280000	-1.2680250000	0.1901400000
C	-1.8397930000	2.2859920000	-0.1918290000
C	-2.1196110000	0.9801750000	-0.0028260000
C	3.8689870000	-1.0416260000	0.2324450000
C	5.1006270000	-1.6856550000	0.3030580000
C	6.2984720000	-0.9667150000	0.2261810000
C	6.2248150000	0.4238570000	0.0686700000
C	4.9992160000	1.0731790000	-0.0032740000
C	0.2191750000	0.8967550000	0.0271730000
C	2.5076440000	1.0817820000	-0.0054170000

C	3.8042460000	0.3475020000	0.0794040000
H	-7.9640450000	-0.9053230000	1.4146980000
H	-7.5036600000	-2.2595140000	0.3860720000
H	-8.1603890000	-0.7911580000	-0.3394790000
H	8.0192270000	-1.6086340000	1.3583440000
H	8.3777850000	-1.1998890000	-0.3180310000
H	7.5552270000	-2.7188740000	0.0681900000
H	-4.0393870000	0.9192610000	-1.8975750000
H	-6.3383410000	0.0515020000	-1.6991470000
H	-5.5500160000	-1.1044920000	2.3534770000
H	-3.2670650000	-0.2164690000	2.1666590000
H	-1.6907250000	-3.6331740000	-0.8988200000
H	0.1414690000	-1.6213340000	-1.6468840000
H	-1.6188170000	-1.5515660000	-1.8526980000
H	-1.8586480000	-1.5995940000	0.6707900000
H	-0.0908640000	-1.5622760000	0.8064310000
H	-2.5620800000	3.0824910000	-0.2732400000
H	2.9492470000	-1.6087580000	0.2907570000
H	5.1326640000	-2.7646900000	0.4168470000
H	7.1411600000	1.0018900000	-0.0006940000
H	4.9443590000	2.1478140000	-0.1272930000

*Table S5: XYZ coordinates for the optimized gas-phase geometry of **1c***

1c optimized geometry (B3LYP/6-311++G(d,p))

S	0.1289820000	-2.6003760000	0.2130290000
F	7.3315270000	1.0815800000	-0.4484720000
F	-7.4397190000	1.5741760000	-0.3255950000
O	0.8020830000	3.3321430000	0.9953570000
O	-2.4678260000	-2.2904930000	0.1716580000
N	0.9644470000	-0.1757460000	-0.1108050000
N	-1.3691290000	-0.2817640000	-0.0867010000
C	3.9259160000	0.2089230000	-1.3094390000
C	5.2197720000	0.7052390000	-1.4282210000
C	6.0715240000	0.6024240000	-0.3381090000
C	5.6777550000	0.0233040000	0.8569540000
C	4.3768410000	-0.4637600000	0.9626660000
C	3.4850420000	-0.3763570000	-0.1134470000
C	0.7824460000	1.9220330000	1.2030440000
C	0.9411460000	1.2905750000	-0.1784930000
C	1.8497560000	-2.2651280000	0.1807630000
C	2.1271020000	-0.9572580000	0.0014160000
C	-3.8573330000	1.0506300000	-0.2741990000
C	-5.0842090000	1.7018570000	-0.3579890000
C	-6.2415370000	0.9461880000	-0.2452310000
C	-6.2201290000	-0.4280570000	-0.0539720000

C	-4.9869840000	-1.0640700000	0.0271120000
C	-0.2099550000	-0.8774630000	-0.0194920000
C	-2.4971680000	-1.0672550000	0.0131070000
C	-3.7958720000	-0.3348460000	-0.0810360000
H	3.2587900000	0.2597210000	-2.1623020000
H	5.5732670000	1.1540970000	-2.3479100000
H	6.3755970000	-0.0379900000	1.6825090000
H	4.0479610000	-0.9145680000	1.8914950000
H	0.6052970000	3.7757960000	1.8253770000
H	-0.1632680000	1.5954060000	1.6476050000
H	1.6090990000	1.6076460000	1.8535300000
H	1.8671520000	1.6373060000	-0.6319190000
H	0.1048960000	1.5825330000	-0.8114040000
H	2.5726170000	-3.0622070000	0.2508030000
H	-2.9363110000	1.6114200000	-0.3598480000
H	-5.1531640000	2.7720130000	-0.5085640000
H	-7.1514410000	-0.9745270000	0.0274080000
H	-4.9264400000	-2.1349560000	0.1751790000

*Table S6: XYZ coordinates for the optimized gas-phase geometry of **1d***

1d optimized geometry (B3LYP/6-311++G(d,p))

S	0.1222580000	-2.7331510000	-0.1263950000
O	7.4364650000	0.8843980000	0.0437000000
O	-7.3629260000	1.6931040000	0.3105450000
O	0.8557540000	3.1360220000	-1.2588600000
O	-2.4691470000	-2.3847970000	-0.0498460000
N	0.9953330000	-0.3061130000	0.0441130000
N	-1.3395020000	-0.3798280000	0.0698700000
C	7.9731100000	1.5678680000	1.1707440000
C	-8.6184790000	1.0263070000	0.2771510000
C	3.9977690000	0.1108540000	1.1384200000
C	5.3004310000	0.6034090000	1.1958140000
C	6.1500100000	0.4435590000	0.0964000000
C	5.6812130000	-0.2128730000	-1.0479550000
C	4.3815320000	-0.6931080000	-1.0927530000
C	3.5127110000	-0.5372470000	-0.0012230000
C	0.8078130000	1.7152650000	-1.3809850000
C	0.9921360000	1.1611390000	0.0300110000
C	1.8485150000	-2.4195710000	-0.1464540000
C	2.1474480000	-1.1078890000	-0.0471830000
C	-3.8101310000	1.0017910000	0.2172420000
C	-5.0217050000	1.6688340000	0.2824900000
C	-6.2236860000	0.9489660000	0.2404730000
C	-6.1947040000	-0.4461340000	0.1330460000
C	-4.9691530000	-1.1030750000	0.0687960000

C	-0.1906870000	-0.9945570000	0.0156590000
C	-2.4827820000	-1.1527320000	0.0363630000
C	-3.7642800000	-0.3972790000	0.1089930000
H	7.4111040000	2.4825430000	1.3866960000
H	7.9851980000	0.9260110000	2.0579040000
H	8.9948990000	1.8276060000	0.9001530000
H	-8.7532800000	0.4752370000	-0.6596150000
H	-8.7288360000	0.3402410000	1.1235860000
H	-9.3719130000	1.8091780000	0.3467220000
H	3.3585380000	0.2201020000	2.0076850000
H	5.6394570000	1.0947510000	2.0976030000
H	6.3516240000	-0.3306710000	-1.8905850000
H	4.0269020000	-1.1943580000	-1.9861210000
H	0.6192070000	3.5310570000	-2.1028000000
H	-0.1545850000	1.3808270000	-1.7813220000
H	1.6123020000	1.3496580000	-2.0322760000
H	1.9341540000	1.5184490000	0.4399990000
H	0.1748610000	1.5003560000	0.6643730000
H	2.5599080000	-3.2288870000	-0.1867460000
H	-2.8816090000	1.5563150000	0.2514300000
H	-5.0671660000	2.7479760000	0.3676190000
H	-7.1091770000	-1.0229010000	0.0994590000
H	-4.9322020000	-2.1824720000	-0.0139360000

*Table S7: XYZ coordinates for the optimized gas-phase geometry of **1e***

1e optimized geometry (B3LYP/6-311++G(d,p))

S	1.2707220000	-2.6268490000	-0.3000930000
O	-7.6893730000	0.1902360000	0.1357640000
O	-6.8373060000	2.1636760000	0.4258730000
O	1.2667050000	3.4866110000	-0.5710390000
O	-1.3539130000	-2.5262400000	-0.3240710000
N	-6.7393890000	0.9561270000	0.2435190000
N	1.8943530000	-0.1486780000	0.0588260000
N	-0.4206530000	-0.4422480000	-0.0095750000
C	4.9179680000	0.6670130000	-0.9132420000
C	6.1504920000	1.3025630000	-0.7867650000
C	6.8987090000	1.1532080000	0.3807750000
C	6.4116800000	0.3623160000	1.4191440000
C	5.1770700000	-0.2728720000	1.2953380000
C	4.4162440000	-0.1237220000	0.1294300000
C	1.4478170000	2.1166470000	-0.9060280000
C	1.7514400000	1.2856680000	0.3473260000
C	2.9562710000	-2.1588030000	-0.1878360000
C	3.1193330000	-0.8329010000	-0.0042090000
C	-3.0062570000	0.6853780000	0.1727570000

C	-4.2814390000	1.2303010000	0.2636830000
C	-5.3787330000	0.3826190000	0.1470180000
C	-5.2381800000	-0.9874540000	-0.0573190000
C	-3.9582160000	-1.5174650000	-0.1462990000
C	0.7873190000	-0.9415260000	-0.0740650000
C	-1.4738140000	-1.3134570000	-0.1361320000
C	-2.8347750000	-0.6889970000	-0.0323330000
H	4.3520030000	0.7642500000	-1.8327390000
H	6.5322580000	1.9037500000	-1.6041160000
H	7.8594940000	1.6457930000	0.4768790000
H	6.9912280000	0.2381920000	2.3267280000
H	4.7962810000	-0.8862230000	2.1038910000
H	2.1176430000	3.8735990000	-0.3410750000
H	0.5089840000	1.7815660000	-1.3458240000
H	2.2420290000	1.9895000000	-1.6513480000
H	2.6790040000	1.6202740000	0.8138070000
H	0.9348420000	1.4046780000	1.0591500000
H	3.7438740000	-2.8885880000	-0.2863660000
H	-2.1344940000	1.3192640000	0.2598320000
H	-4.4362320000	2.2885830000	0.4212920000
H	-6.1183250000	-1.6094100000	-0.1425240000
H	-3.8067920000	-2.5774290000	-0.3051790000

Table S8: XYZ coordinates for the optimized gas-phase geometry of ***1f***

1f optimized geometry (B3LYP/6-311++G(d,p))

S	0.8551320000	-2.7310990000	-0.2858020000
F	7.7924390000	1.4509280000	0.4020990000
O	-8.0051970000	0.3778490000	0.1703820000
O	-7.0888620000	2.3284730000	0.4158690000
O	1.0654540000	3.3666370000	-0.6797210000
O	-1.7621080000	-2.5458490000	-0.2849010000
N	-7.0308580000	1.1151740000	0.2566670000
N	1.5626700000	-0.2706270000	0.0417690000
N	-0.7610750000	-0.4887260000	-0.0065460000
C	4.6391780000	0.3637820000	-1.0018280000
C	5.8896870000	0.9667810000	-0.9017950000
C	6.5772700000	0.8687480000	0.2989530000
C	6.0650980000	0.1902980000	1.3934410000
C	4.8116200000	-0.4062860000	1.2782140000
C	4.0831800000	-0.3243330000	0.0849570000
C	1.2099730000	1.9847530000	-0.9796680000
C	1.4708000000	1.1739650000	0.2965290000
C	2.5564420000	-2.3162040000	-0.1998500000
C	2.7638840000	-0.9950030000	-0.0300330000
C	-3.3087310000	0.7244310000	0.1664030000

C	-4.5653160000	1.3112690000	0.2551460000
C	-5.6896350000	0.4967970000	0.1622190000
C	-5.5941680000	-0.8806560000	-0.0166250000
C	-4.3323460000	-1.4527760000	-0.1040920000
C	0.4288670000	-1.0279600000	-0.0727860000
C	-1.8431050000	-1.3274040000	-0.1146650000
C	-3.1825020000	-0.6583680000	-0.0134120000
H	4.0997340000	0.4112450000	-1.9405760000
H	6.3355840000	1.4928020000	-1.7365990000
H	6.6391550000	0.1330570000	2.3096500000
H	4.3940730000	-0.9417480000	2.1227190000
H	1.9251320000	3.7369660000	-0.4550220000
H	0.2675240000	1.6671780000	-1.4250750000
H	2.0101030000	1.8159560000	-1.7106170000
H	2.4052720000	1.4856990000	0.7652560000
H	0.6522910000	1.3390200000	0.9965780000
H	3.3188270000	-3.0726590000	-0.2961110000
H	-2.4168020000	1.3319370000	0.2353360000
H	-4.6854510000	2.3767680000	0.3931640000
H	-6.4941880000	-1.4757890000	-0.0838810000
H	-4.2159500000	-2.5198360000	-0.2437920000

*Table S 9: Electronic absorption properties of **1a-1f** at the B3LYP/6-311++G(d,p) level of theory in gas phase*

Compounds	λ^{cal} nm	E_{ex} eV	*f*	Orbital Contribution	*λ^{exp} nm
**1a**	315	3.93	0.26	H-L(49%)<br>H-1-L(49%)	339
**1b**	319	3.89	0.36	H-L(57%)	336
**1c**	317	3.91	0.27	H-L(55%)	334
**1d**	320	3.87	0.56	H-L(66%)	338
**1e**	393	3.16	0.21	H-L(71%)	365
**1f**	387	3.20	0.21	H-L(70%)	365

*Table S 10: Electronic absorption properties of **1a**-**1f** at the B3LYP/6-311++G(d,p) and **1e** and **1f** at CAM-B3LYP/6-311++G(d,p) in DMSO*

Compounds	λ^{cal} nm	E_{ex} eV	*f*	Orbital Contribution	*λ^{exp} nm
			**B3LYP**		
**1a**	333	3.73	0.56	H-L(70%)	339
**1b**	334	3.72	0.65	H-L(70%)	336
**1c**	331	3.75	0.57	H-L(70%)	334
**1d**	336	3.69	0.75	H-L(70%)	338
**1e**	461	2.69	0.22	H-L(71%)	365

**1f**	460	2.69	0.22	H-L(71%)	365
			**CAM-B3LYP**		
**1e**	322	3.85	0.63	H-L(60%)	365
**1f**	322	3.85	0.63	H-L(60%)	365

As expected, gas-phase B3LYP values deviate more from experiment, highlighting the importance of solvation [1,2]. When a DMSO continuum was included, B3LYP reproduced the main band for **1a–1d** well ($\lambda_{cal}/\lambda_{exp}$: **1a** 333/339 nm; **1b** 334/336 nm; **1c** 331/334 nm; **1d** 336/338 nm). In contrast, for the nitro-substituted analogs **1e** and **1f**, B3LYP/C-PCM markedly overestimated λ_{cal} (by ~95–96 nm), consistent with known difficulties of conventional hybrids in describing low-lying charge-transfer excitations [3,4]. Since the elevated long-range Hartree-Fock exchange in CAM-B3LYP (65% vs 20% in B3LYP) reduces the self-interaction error that destabilizes CT states, making it better suited for systems containing strongly electron-withdrawing groups, such as nitro groups [5], CAM-B3LYP/C-PCM was used for **1e** and **1f**, yielding λ_{cal} ≈ 322 nm for both compounds (**Table S10**).

References

[1] J. Ge, X.D. Zhang, Y. Peng, X.L. Bai, Unraveling the effect of solvents on the excited state dynamics of C540A by experimental and theoretical study, *RSC Adv.* 13 (2023) 4924–4931. https://doi.org/10.1039/d3ra00259d

[2] O.M.I. Adly, A. Taha, S.A. Fahmy, M.A. Ibrahim, TD-DFT calculations, dipole moments, and solvatochromic properties of 2 -aminochromone-3-carboxaldehyde and its hydrazone derivatives, *RSC Adv.* 13 (2023) 26587–26603. https://doi.org/10.1039/d3ra05081e

[3] H. Nitta, I. Kawata, A close inspection of the charge-transfer excitation by TDDFT with various functionals: An application of orbital- and density-based analyses, *Chem. Phys.* 405 (2012) 93–99. https://doi.org/10.1016/j.chemphys.2012.06.016

[4] C.R. Zhang, J.S. Sears, B. Yang, S.G. Aziz, V. Coropceanu, J.L. Brédas, Theoretical study of the local and charge-transfer excitations in model complexes of pentacene-C60 using tuned range-separated hybrid functionals, *J. Chem. Theory*

Comput. 10 (2014) 2379–2388. https://doi.org/10.1021/ct500259m

[5] C. Halsey-Moore, P. Jena, J.T. McLeskey, Tuning range-separated DFT functionals for modeling the peak absorption of MEH-PPV polymer in various solvents, Comput. Theor. Chem. 1162 (2019) 112506. https://doi.org/10.1016/j.comptc.2019.112506.
